# Supplementary material for: Attenuated PDGF signaling drives alveolar and microvascular defects in neonatal chronic lung disease
Source: EMBO Mol Med. 2017 Sep 18;9(11):1504–20. doi: 10.15252/emmm.201607308 (PMC5666314; doi:10.15252/emmm.201607308)
Supplement: Supplementary file 1 — Appendix [file EMMM-9-1504-s001.pdf]

TABLE OF CONTENTS

APPENDIX SUPPLEMENTAL METHODS* _______________ *2

Study Design _______________ 2

Human studies _______________ 2

***In vivo* studies _______________ 7**

***In vitro* experiments _______________ 9**

APPENDIX FIGURES AND FIGURE LEGENDS* _______________ *13

APPENDIX TABLES* _______________ *20

APPENDIX REFERENCES* _______________ *26

APPENDIX SUPPLEMENTAL METHODS

Study Design

Preterm infants born below 32 weeks of gestational age and matched control infants with respect to gestational age, gender, percentage of individuals considered small for GA, and country of maternal origin were selected for the study. Exclusion criteria were oligo- or anhydramnios, severe congenital malformations, severe metabolic disorders and prepartum treatment of the mother with cytostatic or immunosuppressive medication other than for lung maturation.

Mice were randomly selected from each litter to undergo mechanical ventilation. 6-11 mice were utilized per group for histological analysis as well as 3-6 mice per group for protein analysis. Tissue sections from the respective mouse lungs were selected randomly for hematoxylin and eosin as well as immunostainings. *In vitro* experiments were performed on myofibroblasts obtained from 3-9 different mice. Functional assays on both mice myofibroblasts and human lung fibroblasts (proliferation and migration analysis) constituted 3 biological replicates consisting of 2-3 technical replicates each resulting in a total of 5-9 samples from different mice analyzed per condition (stretch and/or TGF-β treatment).

Human studies

SNP and protein analysis. All relevant perinatal diagnoses were provided for statistical analysis. Patients with moderate or severe BPD according to the definition by Jobe et al. from both cohorts were considered cases (Jobe, 2011). Exclusion criteria were oligo- or anhydramnios, severe congenital malformations, the diagnosis of severe

metabolic disorders and prepartum treatment of the mother with cytostatic or immunosuppressive medication other than for lung maturation led to exclusion of the neonate. Cases (controls) had a median GA of 26.6 (27.0) weeks, 42.5 (45.7) % were female, and 28.0 (26.7) % were found to be small for GA. Duration of oxygen supplementation was 80 (15) days in median. Genotypes of PDGF-Rα SNPs (single nucleotide polymorphisms) were determined in neonates using Affymetrix Axiom microarrays based on the Axiom CEU array supplemented with some custom content. Among the 1061 individuals with genetic data passing QC, 492 developed BPD grade 2 or 3 and were considered cases. Cases and controls were balanced for sex, gestational age at birth, status "small for gestational age", and country of origin of the mother. 117 SNPs within 100 kB upstream and downstream of PDGF-Rα, present on the array, and passing QC were selected for analysis; case-control analysis was adjusted for relatedness to account for multiple births. The PDGF signaling pathway and several pathways downstream of PDGF signaling were analyzed for evidence regarding genetic regulation of gene expression of their pathway elements. From our genome-wide dataset, genetic markers were identified either directly or via linkage disequilibrium as corresponding to SNPs found to contribute to genetic regulation of gene expression (i.e. eQTL SNPs) in several studies (Ding et al, 2010; Dixon et al, 2007; Fehrmann RS, 2011; Xia et al, 2012; Zeller et al, 2010). The distribution of p-values for these SNPs was analyzed using the R-package snpMatrix (Devlin & Roeder, 1999) to identify pathways for which p-values in our BPD-association analysis showed nominally more evidence for association than would be expected by chance. A part of patients from this

cohort (n=9, Appendix Table S3A) were subjected to PDGF-Rα transcriptome analysis as described in microarray methods section.

A separate patient cohort consisting infants with or without later development of BPD and a GA <32 weeks with excluding growth retardation was included at the Perinatal Center of the Ludwig-Maximilians-University, Campus Grosshadern (n=13). Patient characteristics of this cohorts are given in **Appendix Table 3B**. Whole blood samples were collected at days 22-58 after birth in Ethylenediaminetetraacetic acid (EDTA) neonatal collection tubes. Generated plasma samples were stored at -80 °C and later subjected to proteomic screening (SOMAscanTM, SomaLogic, Boulder, USA). For the assay high quality samples were used and protein binding to 1129 individual high affinity molecules (SOMAmer®) was quantified by custom Agilent hybridization array (Rohloff et al, 2014). Even low amount samples (90-160µl) showed high reproducibility. The SNP genotyping of this samples for the most significant SNP position rs12506783 was performed by Eurofins Genomics. Briefly, sequences forward CAAAATACCTGGAAGCTCTGGAG and reverse CCAGCATTCAATTCATACTTGCTG were generated using a re-sequencing approach. According to the reference sequence, the necessary primers were defined in order to create overlapping PCR fragments and for subsequent sequencing to have the region of interest covered in a satisfactory manner. PCR purification was performed with 10% PolyEthylenGlycol (PEG8000) in 30% Isopropyl alcohol/1M NaCl for precipitation and a washing step with 80% Ethanol. All sequences were generated using BigDye terminator chemistry (version 3.1), if necessary in combination with dGTP BigDye terminator chemistry (version 3.0) (Thermo Fisher Scientific, Waltham, MA USA) following standard protocols. For sequencing

reactions peqStar 96 HPL (PEQLAB Biotechnologie GMBH, Erlangen, Germany) or GeneTouch (Biozym Scientific GmbH, Oldendorf, Germany) thermal cyclers were used. Sequencing reaction cleanup was done either manually or on a Hamilton Starlet robotic workstation (Hamilton Robotics GmbH, Martinsried, Germany) by gel-filtration through a hydrated Sephadex matrix filled into appropriate 96well filter plates followed by a subsequent centrifugation step. Finally all reactions were run on ABI3730xl capillary sequencers equipped with 50 cm capillaries and POP7 polymer (Thermo Fisher Scientific, Waltham, MA USA). Sequencing data was generated using the original ABI Software including the KB-basecaller, which assigns quality values to all called bases similar to PHRED (Ewing et al, 1998). Additional basecalling was performed using the PeakTrace basecaller from Nucleics Pty Ltd (Woollahra, AUS) according to SOP_SEQ_PeakTrace to improve the single peak resolution and quality values and therefore increase the reading lengths. Primer sequences used were forward GTACTGGGATTACAGGTGTGAG and reverse ATAACATCCCAGGAGGCCTAC.

Human primary lung fibroblasts. Patients for tracheal aspirate analysis were recruited following the same in- and exclusion criteria as outlined above. Samples from 6 patients were used. Two patients provided serial samples; two patients were recruited with individual samples with respect to the early and later time point for PDGF-Rα expression analysis. Tracheal aspirates were obtained from preterm infants undergoing MV-O_2 who later developed nCLD, i.e. mild, moderate or severe BPD (gestational age 24 ± 1.4 weeks, birth weight 650 ± 80.2 g, MV-O_2 43 ± 24 days (mean ± SD)). Patient characteristics are mentioned in **Appendix Table S3C**. First samples were obtained early after the initiation of MV-O_2 (4.7±1 day of life); second samples were taken during

the third week of life (21.7±8 day of life). Primary human lung fibroblasts were cultured 80% confluent in DMEM medium with 20% FCS (PAN Biotech GmbH, Aidenbach, Germany), 2mM L-glutamine, and penicillin/streptomycin (Thermo Fisher Life Technologies, Carlsbad, CA). FACS analysis was performed to check the purity of the cultures as described in the manuscript.

Blood sampling, RNA isolation and microarray analysis. Briefly, blood was sampled from an indwelling umbilical artery catheter at birth. RNA isolation was performed according to the manufacturer's recommendations (PreAnalytiX). RNA was hybridized on CodeLink Human Whole Genome Bioarrays (GE Healthcare) using the CodeLink Expression Assay Kit (GE Healthcare) and samples processed using CodeLink Expression Software V4.1 (GE Healthcare). The dataset was initially prepared using the manufacturer recommended subtract background correction and median normalization. Data was then filtered for transcripts with high rates of missing values, low expressed values, or outlier values. Missing values were imputed using the sequential nearest neighbor (SeqKNN) approach (Kim et al, 2004). The dataset was then normalized using the quantile normalization method (Bolstad et al, 2003). For data preparation the Bioconductor packages SeqKnn and limma for quantile normalization were used.

Human lung slides. Human slides were obtained from paraformaldehyde fixed and paraffin embedded autopsy lungs from preterm infants with different BPD grades (n=7) and an infant that died from a non-pulmonary cause. Median gestational age of BPD patients was 26+0 weeks (25+4 – 31+0) and gestational age of the control infant was 26+5 weeks. All patients died between 28 and 66 days after birth and none were

treated with extracorporeal membrane oxygenation. No chromosomal or other congenital anomalies were present in all patients. Tissue sections were stained for PDGF-Rα, and TGF-β for further quantification. The patient characteristics are mentioned in **Appendix Table S3D.**

***In vivo* studies**

Assessment of protein expression in distal lung. PFA-fixed lung tissue sections were stained for PDGF-Rα (C-20) (Santa Cruz Biotechnology #sc-338), α-SMA (Sigma Aldrich # A5228), VEGF-A (C-1) (Santa Cruz Biotechnology #sc-7269), CD31 (Dianova, Hamburg Germany #DIA-310), cleaved caspase-3 (Cell Signaling Technology #9661S), VE-Cadherin (H-72, Santa Cruz Biotechnology #28644), pSMAD-2/3 (Santa Cruz Biotech #SC-8828) and DAPI (Sigma Aldrich #D8417) alone or in combination. Number of total nuclei as well cleaved caspase-3 and CD-31 positive cells were quantified in eight different fields of view/slide (400x magnification) using Imaris Software (Zurich, Switzerland). For cell surface or extracellular markers, areas with positive stain were quantified in separate color channels in 10 fields of view/animal (200x magnification) using the BIOQuant Software (BIOQUANT Image Analysis Corporation, Nashville, TN, USA).

Protein extraction and immunoblot analysis. Lungs from 8h studies (n=5-8/group) were excised, weighed and snap-frozen in liquid N_2, and stored at -80°C for later protein extraction. Protein extraction was performed using high urea buffer (KPO_4, Urea, AppliChem, Darmstadt, Germany) with added Halt Protease Inhibitor Cocktail (catalog #1861280, Thermo Fisher Scientific). Measurement of protein concentrations

was done using the bicinchoninic acid (BCA) assay (catalog #23227, Pierce Scientific Rockford, IL, USA) and immunoblots were performed using a Bis-Tris (catalog #NP0321BOX, Life Technologies, Darmstadt, Germany) or a Tris-Acetate (catalog #EA0375BOX, Life Technologies) Gel according to the manufacturer`s instructions. After protein transfer (Nitrocellulose/Filter Paper, catalog #LC2006, Life Technologies) and blocking using 5% skim milk (catalog #70166, Sigma Aldrich) in 0,1% TBS-T buffer, the membranes were incubated with the following antibodies at 4°C overnight: PDGF-Rα (C-20, Santa Cruz Biotechnology #338), VEGF-A (147, Santa Cruz Biotechnology #507), VEGF-R2 (Abcam, Cambridge, USA #Ab2349), VE-Cadherin (H-72, Santa Cruz Biotechnology #28644), cleaved caspase-3 (Cell Signaling Technology #9661), phospho-ERK (Cell Signaling Technologies #4370), total ERK (Cell Signaling Technologies #4695), RAS (Cell Signaling Technologies #8955), PI3K (Cell Signaling Technologies #13666). After washing the membranes were incubated with secondary antibodies (1:5000 dilution) as follows: for PDGF-Rα, VEGF-A, VE-Cadherin, cleaved caspase-3, phosphor ERK, total ERK, RAS, PI3K: goat anti-rabbit IgG (Santa Cruz Biotechnology #2301) and for VEGF-R2: donkey anti-goat IgG-HRP (Santa Cruz Biotechnology #2020) conjugated to horseradish peroxidase for 1-2 hours at 4°C followed by 3 washes. As an internal loading control, membranes were stripped and re-probed with 1:5000 dilution of a mouse polyclonal anti-β-actin antibody (Santa Cruz Biotechnology #sc-81178) followed by 1:5000 dilution of goat anti-mouse IgG-HRP (Santa Cruz Biotechnology #2060). Images were detected by chemiluminescence ECL prime Detection Kit (GE Healthcare, Buckinghamshire, Great Britain #RPN2232) and

quantified by densitometry using a Gel Documentation System (Bio Rad, Munich, Germany).

RNA extraction and quantitative real-time PCR. Lungs from 8h studies (n=4-5/group) were excised, weighed, snap-frozen in liquid N_2, and stored at -80°C for subsequent two-step mRNA extraction using Roti-Quick-Kit (Carl Roth GmbH #A979.1) and purification with peqlab-Gold Total RNA-Kit (Peqlab, Erlangen, Germany #12-6834-01). Quantitative real-time PCR was applied to measure lung mRNA expression of PDGF-Rα (forward 5'-TGTGCCGTTTCTCACTTCTCCAG-3', reverse 5'-TACCTTTGTTTCTCACTTCTCCAG-3') using proprietary primer-probes (Eurofins mwg operon, Ebersberg, Germany).

In vitro experiments

Mechanical stretch experiment Human lung fibroblasts and mouse myofibroblasts were seeded on flexible-bottomed laminin-coated culture plates (Flex Cell International Corporation catalog no.: BF-3001L) to undergo *in vitro* stretch at 70-80% confluence (cyclic strain by vacuum pressure: shape / sine; elongation min 0%, max 8%; frequency 2Hz; duty cycle 50%; cycles 43216; duration 24h) for 24h. As shown in **Appendix Figure S3C,** one set of myofibroblasts was kept as un-stretched untreated control (C). Myofibroblasts undergoing stretch for 24h were termed as S and those undergoing stretch with parallel application of 5ng/ml TGF-β for 24h were termed as S+Th1. A set of myofibroblasts was also subjected to 5ng/ml TGF-β alone i.e. without any additional application of stretch and was termed as Th1. This step was considered as first hit on

the myofibroblasts. A part of pre-stretched myofibroblasts (S+Th1) was separated from group and re-incubated (second hit) with 5ng/ml TGF-β (S+Th1+Th2). The dose of TGF-β and stretching parameters caused the injury without inducing apoptosis in myofibroblasts (**Appendix Figure S4A-B**). Apoptosis was detected using Annexin V FITC stain (BD Pharmingen #51-65874X) and Propidium Iodide stain (Sigma Aldrich #P4864) as per manufacturer's instructions and the data was analyzed using fluorescence antibody cell sorting device (FACS LSRII). Viability was determined using luminescence based Cell titer glo assay (Promega GmbH, Germany #G757) as per manufacturer's instructions and reading were taken with Berthold multimode microplate reader LB 941 (Berthold Technologies GmbH, Germany)

Generation of reporter constructs and Luciferase assay The mouse PDGF-Rα promoter construct spanning from -1074 to +280 from initiation of transcription manufactured by gene synthesis (Life Technologies) was cloned into pGL4.14 (Promega, Madison, WI). Construct sequence was confirmed by sequencing (**Appendix Figure S5**). CCL206 cells were stimulated with TGF-β1 1 ng/ml (Peprotech, Rocky Hill, NJ) followed by transfection (TurboFect, ThermoFisher) with reporter plasmids (1 µg) and PGK Renilla (50 ng, Promega). Luciferase activity was determined using the Dual Luciferase Assay (Promega) the next day and values of Firefly Luciferase were normalized to values of Renilla Luciferase. The baseline reporter activity of unstimulated cells was set to 1.0. α-smooth muscle actin expression was determined as positive control read-out to prove TGF-β1 activity using Western blot analysis 48 hours later.

Proliferation assay After first hit of stretch with/without TGF-β as shown in supplemental figure 2, mouse myofibroblasts and human lung fibroblasts were seeded

in a 24 well plate (75000 cells) for manual counting or in 96 well plates (5000 cells/well) for luminescence based Cell titer glo assay while performing second hit of TGF-β keeping respective controls. For manual counting myofibroblasts were trypsinized after 48h of incubation with second hit of TGF-β and counted using Neubauer chamber. Cell titer glo assay was performed as per manufacturer's instructions after 48h of incubation with second hit of TGF-β (Cell Titer-Glo assay kit; Promega GmbH, Germany, #G7571). Briefly, reagent equivalent to the amount of media in each well was added. Plates were shaken gently and kept at 37°C for 10 min followed by reading the plate using plate reader (Berthold technologies Tristar LB 941, Bad Wildbad, Germany).

Boyden chamber or transwell migration assay Cell culture inserts i.e. transwells (8 µm pore size), were placed in wells of 24 well plates containing media (10% FCS) either with 25ng/ml PDGF-A (control) or 25ng/ml PDGF-A and 5ng/ml TGF-β. PDGF-A acted as chemoattractant for migration of myofibroblasts. After 1st hit of stretch and/or TGF-β (**Appendix Figure S3C**), myofibroblasts were seeded in the wells as described above followed by incubation for 8h at 37°C. After washing off the media, myofibroblasts on the upper side of the membrane were scratched and removed. Migrated myofibroblasts on the lower side of the membranes were then fixed in Methanol for 20 min at -20^{0}C and stained with 0.5µg/ml DAPI solution (Sigma Aldrich #D8417) for 10 min. After incubation with 4% PFA for 15 min, cut membranes were mounted with Fluorescent mounting media (Dako North America Inc. #S3023). Images were taken with fluorescence microscope and quantified using Imaris 8.1 software.

Scratch migration assay Human lung fibroblasts were seeded in a 24 well plate and were grown until confluency of 90%. A scratch was prepared in the middle of the

well followed by 5ng/ml TGF-β application keeping respective control. Images were taken every 10 min for 48h using a time lapse microscope. Quantitative analysis of velocity and distance accumulated by the cells was performed using ImageJ as well as chemotaxis and migration softwares.

APPENDIX FIGURES AND FIGURE LEGENDS

Appendix Figure S1.

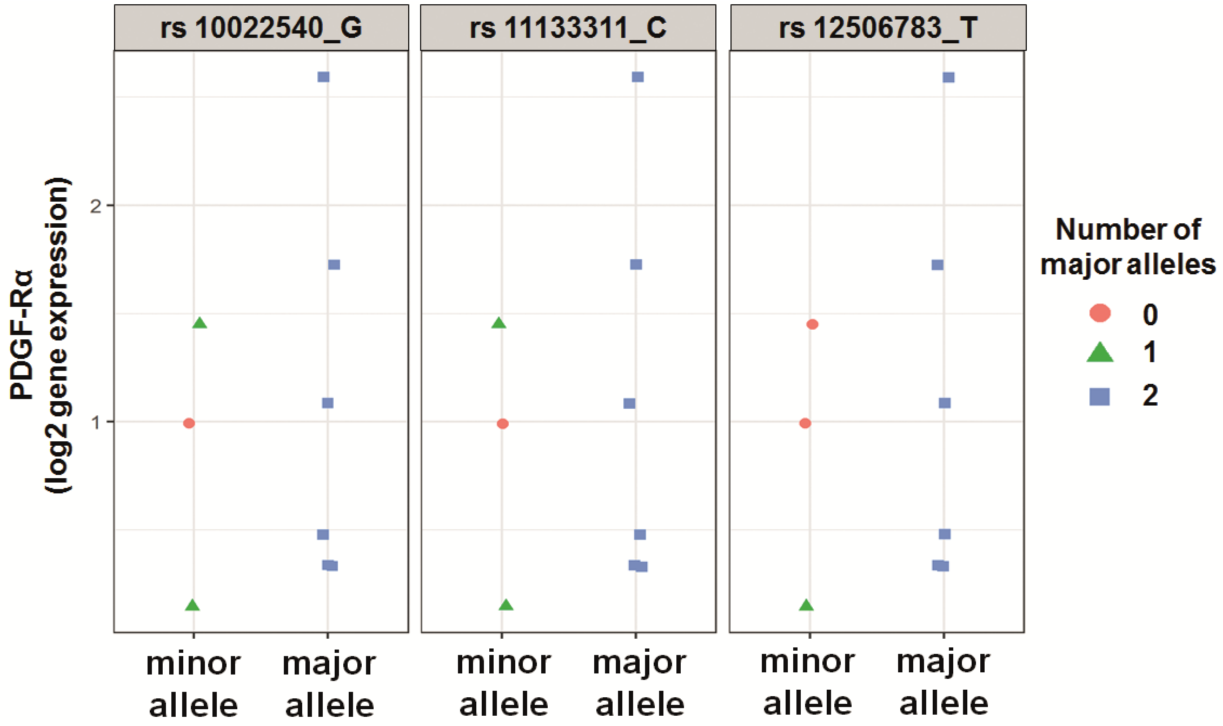

Appendix Figure S1: Gene expression data based on SNP analysis in nCLD patients. PDGF-Rα gene expression in patients (n=8) that carry at least one SNP (minor allele) compared to patients with no SNPs (homozygote major allele). Major alleles are given in the figure labels. Minor alleles of rs10022540 is A, in rs11133311 is T, and in rs12506783 is C. 0 represents homozygote minor allele, 1 represents heterozygote minor allele and 2 is homozygote major allele.

Appendix Figure S2.

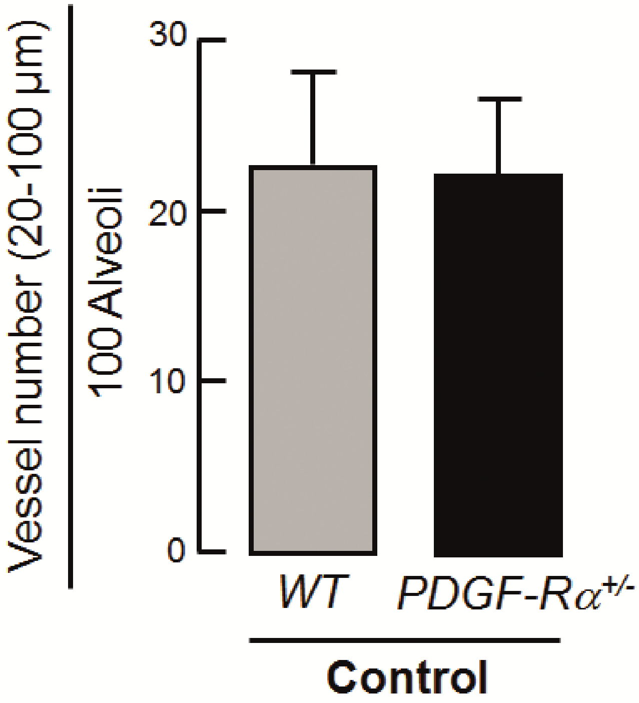

Appendix Figure S2: Vessel count in wildtype and PDGF-Rα$^{+/-}$ mice. Histological analysis showed similar small-vessel number (20-100μm diameter) normalized to 100 alveoli in both unventilated wildtype and PDGF-Rα$^{+/-}$ mice. (n=10-11mice/group).

Appendix Figure S3.

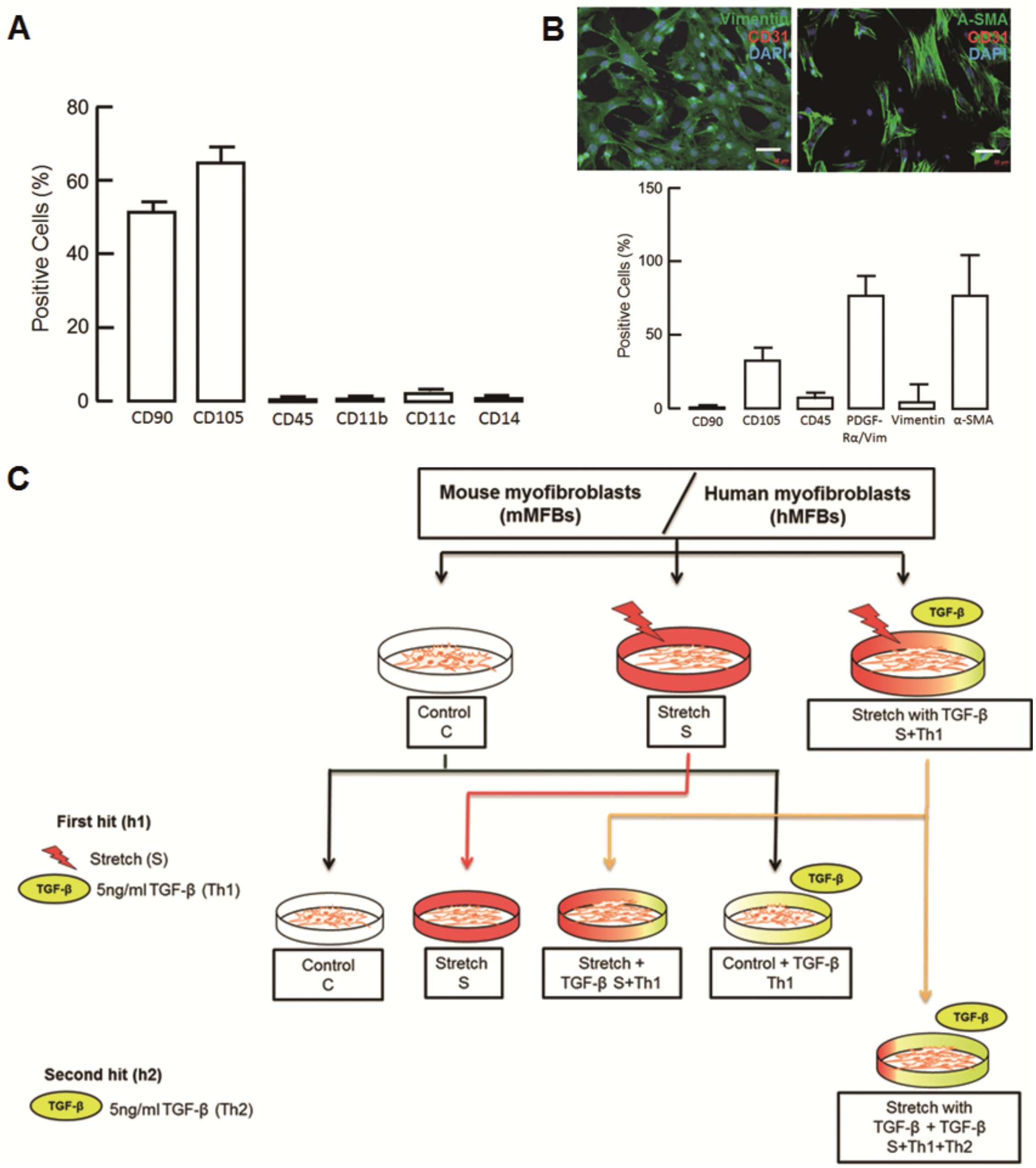

Appendix Figure S3: Characterization of human lung fibroblasts and mouse myofibroblasts and experimental design for *in vitro* studies.

(A-B) Quantitative analysis of characterization of **(A)** human lung fibroblasts and **(B)** mouse myofibroblasts performed using fluorescence antibody cell sorting (FACS) analysis indicated abundance of myofibroblast markers (CD90, CD105, PDGF-Rα, α-SMA and Vimentin). Data is displayed as percentage positive cells. For mouse myofibroblasts n=6mice/group and for human lung fibroblasts. (n=5 samples/group).

(B) Immunofluorescence images (200X) of mouse myofibroblasts co-stained for Vimentin or α-SMA (green), CD31 (red) and nucleus stained with DAPI (blue) are shown in figure.

(C) Schematic representation of experimental strategy used for primary pulmonary mouse myofibroblasts and human lung fibroblasts. Briefly myofibroblasts underwent *in vitro* stretch with or without additional 5ng/ml TGF-β incubation for 24h (S+Th1 and S respectively). A set with only TGF-β application (Th1) and an un-stretched untreated control (C) was performed. This was considered to be the first hit model. A part from S+Th1 Myofibroblasts was re-incubated with 5ng/ml TGF-β for 24h (S+Th1+Th2). This was considered to be second hit model.

In (A-B) the data is presented as mean ± SD. Statistical test is Two-tailed unpaired Student's t test or Mann Whitney test.

Appendix Figure S4.

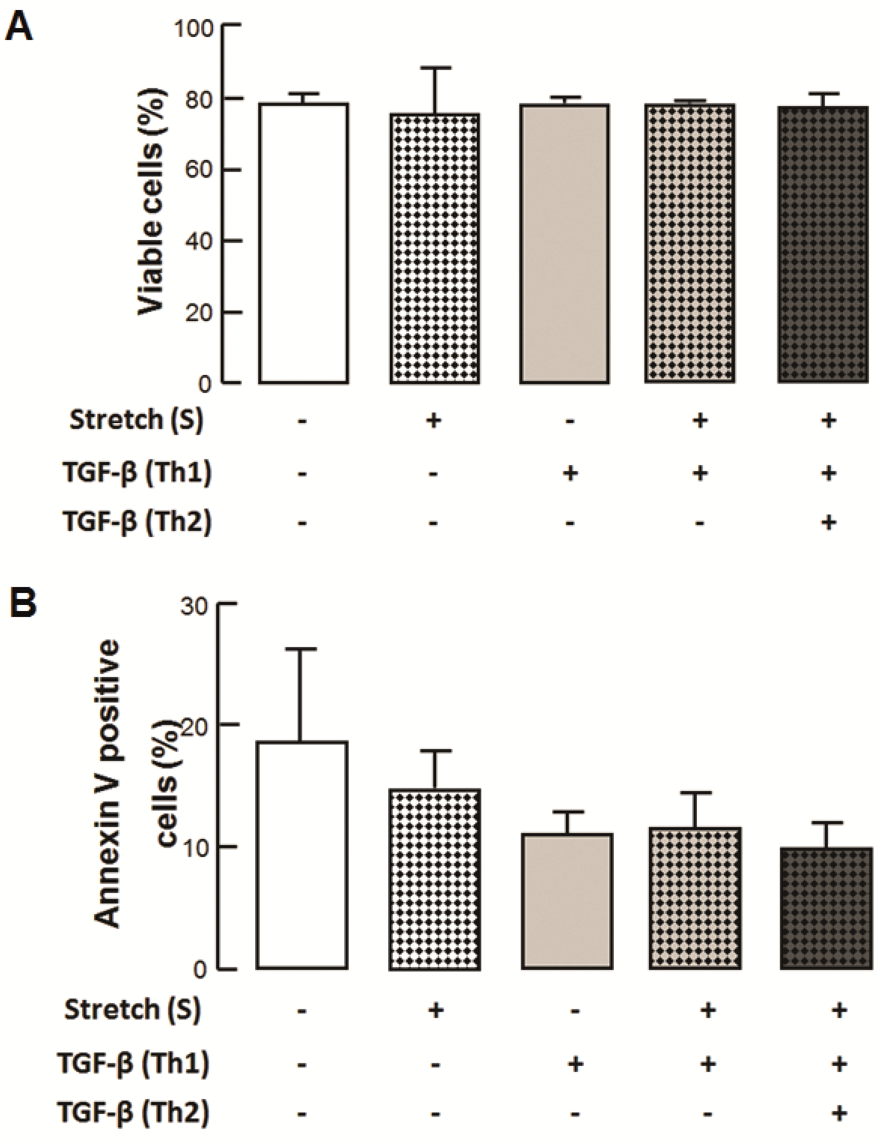

Appendix Figure 4: Viability of mouse myofibroblasts upon application of stretch and TGF-β.

Mouse myofibroblasts subjected to mechanical stretch with or without additional application of TGF-β as shown in supplemental Figure 3. (**A**) For viability analysis Cell

titer Glo assay was performed as per manufacturer's instructions. Luminometric readings displayed approximately 80% viable cells after all the applications for 24h-48h. n=3 mice/group.

(B) Annexin V FITC and propidium iodide staining of mouse myofibroblasts subjected to mechanical stretch with or without additional application of TGf-β as shown in Supplemental figure 3, for analysis of apoptosis. FACS analysis revealed 10-15% apoptosis upon application of stretch with/without TGF-β application as first as well as second hit. (n=3mice/group).

In (A-B) the data is presented as Mean ±SD.

Appendix Figure S5.

```
CATTCAAAAATAGAGCGCAAGTCGAAGGCCCTCCCTTCCCCCACCCGCTCCGGGAAGTCCCGCTTCGCCCAGGTT
TGGTTCCTGGAGTGTCAGCGCCCCCTTTCCCTCGCCAGCAGGATCGCCGTGTCCCAACTGTCTCTCGCTGGGTGT
CTTGGGTTCCCTGGTTTGTGGCTCAAACGTCCTGAAAGCTTTCCTAGGGTACGTCGCGTGCGACCCACTCGGAAG
GGTGGAATTTAGGAGGATAAAAATCCTTCTGCCATCAAGATGCAGAGGGCAGGCATTTGGTAGTCACGCCTAGCC
TGAGCGTTTTCATATGAAGATAGAAGAAGCGAGGACCAGATAACCCCCGAAAACAAAGGCAGGACCAGATAAGTG
GCTCCGAAGGGATAAAGGTCGTTCTTTCCTTCTTCCGGAGAACATCCCAAAGGTACGCCGAGCAACCGTTATTTG
CACACCGCCTCACAATCCAGCCTTTCAAAAACCCATCATCTTCCTATTAGACTCCACAGTTTCCTAATCCCATTA
AAGGATTAGCAACTACACGGCACTTTCCCTTAAGACCCCCAGTTCAAAACGACGCAGCCGCGTTAGAATTTCTCC
CCAGGGCCATATTTCTAGCGAGGCCCAGACTGTCTATAGAAAGGATAATTTGAATTCTAGATTTATATTCGTTTT
AGAAATGAGCAGCAATTTTACGTGTATTTTCTTTTCGAAGAGAATACAAAACAATAGCACCCCCACCCCCAAATT
GGGAAGTCAACTCATTTTGGAAATGATGGCTGTTTGTAGTTTCCTGAAACCTCTTTCCCGGCAGAACGTCAACAC
CTCCCCCTTCGGCCCCCCACCCCACCCCATCTGGTTTGCTCCCCCCTCCTCGTTGTTGTTGAAGTCTGGGGGTTG
GGACTGGCCCCCCTGATTGCATAAGAGCAAAAAGCAAAGAAGAGGTCTTGAGCCTGAGAGAGTCAGAGAGCAAGG
AGTCCTAGGGAAACTTTTATTTTGAAGAGACCAAGGGGGGGGGGACTTCATTTCCTGACAGCTATTTACTTTAA
GCAAATGATTAGTTTTTGGAGGACGGACTATAACATTGAATCAATTACAAAATGCGGGTTTTGAGCCCATTACTG
TTGGAGCTTGAGGGAGAGAAACAAACGGAGGAGCTGCGGGGAAGGACTGGAAGCTTGGGGCTTACTTTTCACTCC
GGGTATCGGATTTTCTTTGCAAATTGACATAGAAGGAGAAGGTAAGGGAGAGGAAAAAGTGACTTTTGTTTCTCA
AGAAGGTCCCTGATCATAACTTGGGGCTGCAAGAAGCTAAGTAACTTCAAATTTTGGGCAACGAGGAAAACAAAA
ACAA
```

Appendix Figure 5. Sequence of gene cloned in pGL4.14 plasmid for performing luciferase assay.

APPENDIX TABLES

Appendix Table S1.

rs-number	Chr	Position	Allele A	Allele B	N homo minor	N hetero	N homo major	N missing	beta	SE	p-value
rs10010509	4	54851963	T	G	18	245	795	3	0,1034	0,1251	0,4089
rs10022540	4	54739808	A	G	174	518	369	0	-0,3038	0,0899	0,0008
NA	4	54739883	A	G	266	509	275	11	-0,2333	0,0839	0,0055
rs1004564	4	54937382	A	C	322	522	217	0	0,1206	0,0885	0,1734
rs11728027	4	54896943	A	G	144	489	425	3	0,0429	0,0902	0,6348
rs11735716	4	54896828	T	C	43	379	634	5	-0,1443	0,1045	0,1679
rs11737133	4	54717553	T	C	152	475	433	1	0,2192	0,0894	0,0144
rs12233727	4	54768027	T	C	248	554	258	1	-0,2826	0,0898	0,0017
rs12500279	4	54775074	T	C	94	444	523	0	0,256	0,0964	0,008
rs12511976	4	54857028	T	C	893	160	7	1	-0,1854	0,1605	0,2484
rs13118725	4	54690466	T	C	630	364	67	0	-0,2356	0,1018	0,0208
rs1492769	4	54952368	A	C	747	285	29	0	-0,1425	0,1195	0,2333
rs17084051	4	54782338	A	C	36	329	696	0	0,123	0,1135	0,2789
rs17084241	4	54946036	A	G	9	146	905	1	0,1937	0,1586	0,2222
rs17690091	4	54924101	T	C	48	380	632	1	0,1779	0,1065	0,095
rs17690232	4	54929582	C	G	697	324	40	0	-0,189	0,1119	0,0917
rs17739921	4	54859623	A	C	329	526	202	4	-0,1739	0,0883	0,0492
rs17746992	4	54932752	C	G	40	325	696	0	0,1951	0,1119	0,0815
rs1907814	4	54940014	A	G	78	461	521	1	-0,0493	0,0994	0,6202
rs1994810	4	54924241	A	G	67	435	559	0	-0,095	0,1021	0,3523
rs2412564	4	54934704	A	G	248	527	286	0	0,1359	0,0881	0,1236
rs35597368	4	54834528	T	C	858	191	12	0	-0,0213	0,1457	0,8839
rs4572929	4	54940498	A	G	68	418	573	2	0,2292	0,1	0,0222
rs4864510	4	54883219	T	C	370	501	188	2	-0,0968	0,0881	0,2725
rs6811920	4	54945988	C	G	168	505	386	2	-0,0349	0,0894	0,6964
rs6832891	4	54751401	T	C	587	412	62	0	-0,1216	0,1032	0,2386
rs6840522	4	54725132	A	G	795	247	19	0	-0,021	0,1297	0,8717
rs7656613	4	54836600	T	C	564	423	74	0	0,2	0,1002	0,0461
rs7659654	4	54731166	T	C	536	429	95	1	-0,2163	0,0958	0,0241
rs7660560	4	54829151	A	G	17	209	833	2	0,1542	0,1323	0,2442
rs7684220	4	54893055	T	C	351	517	186	7	-0,0898	0,088	0,308
rs894905	4	54916706	T	C	251	539	270	1	0,0684	0,0889	0,4419
rs9991165	4	54819348	A	G	791	247	23	0	-0,1153	0,1267	0,3629
rs2228230	4	54846797	T	C	19	250	792	0	0,0673	0,1294	0,6032
rs3690	4	54856570	A	C	798	244	19	0	-0,0888	0,1301	0,4951
rs56404781	4	54698999	T	G	793	251	17	0	0,0151	0,1308	0,9081
rs7378056	4	54739260	A	G	63	413	584	1	0,1423	0,1021	0,1638

rs34148754	4	54751806	A	G	52	415	591	3	0,1304	0,1034	0,2075
rs12506783	4	54781154	T	C	244	541	275	1	0,3076	0,0886	0,0005
rs61320297	4	54782932	A	G	37	328	695	1	0,1306	0,112	0,2438
rs7673984	4	54783518	T	C	36	327	697	1	0,1073	0,1125	0,3404
NA	4	54784627	A	C	871	181	9	0	0,0443	0,1519	0,7708
rs2114039	4	54787383	T	C	583	403	74	1	-0,0667	0,1	0,5049
rs7678144	4	54797182	T	C	696	328	37	0	-0,0953	0,1132	0,3999
NA	4	54810805	C	G	0	139	915	7	-0,0824	0,1527	0,5896
rs7677751	4	54819217	T	C	17	219	824	1	0,0353	0,1331	0,791
rs73252946	4	54820749	A	G	22	243	796	0	0,117	0,1279	0,3605
rs58435984	4	54822747	T	C	834	209	17	1	-0,113	0,1364	0,4078
rs55947416	4	54824588	T	C	5	152	903	1	0,2635	0,1632	0,1067
rs67279506	4	54825439	A	G	835	209	16	1	-0,0655	0,1374	0,6337
rs58727676	4	54825551	T	G	837	207	17	0	-0,088	0,1372	0,5212
rs7688997	4	54826207	A	C	17	209	834	1	0,1276	0,1345	0,3431
rs56145315	4	54826446	T	C	17	209	835	0	0,1039	0,1369	0,4481
rs28600756	4	54826489	A	C	21	205	833	2	0,0951	0,1296	0,4635
rs67600360	4	54826919	A	G	834	209	17	1	-0,1168	0,1364	0,3921
rs28650939	4	54828716	T	C	17	207	837	0	0,1054	0,1372	0,4424
rs28528897	4	54829096	C	G	834	209	17	1	-0,0956	0,1364	0,4834
rs7691129	4	54829223	T	C	835	209	17	0	-0,1039	0,1369	0,4481
rs12641563	4	54830204	C	G	834	209	17	1	-0,0967	0,1364	0,4787
rs12506290	4	54830889	A	T	17	210	828	6	0,0818	0,1245	0,5112
rs10028020	4	54838334	A	G	11	186	862	2	0,0095	0,1423	0,947
rs4289498	4	54840189	A	G	775	256	22	8	-0,1087	0,1235	0,379
rs1547905	4	54841511	A	C	20	246	794	1	0,0349	0,1272	0,7838
rs2291591	4	54842526	T	C	7	158	894	2	-0,0988	0,1543	0,5219
rs7677708	4	54844015	A	G	784	254	22	1	-0,1039	0,1263	0,4108
rs55732997	4	54849648	T	C	792	249	20	0	-0,0755	0,1287	0,5578
rs10004857	4	54850737	A	G	18	244	796	3	0,0354	0,1253	0,7773
rs11133317	4	54850848	T	G	20	249	792	0	0,1086	0,1287	0,3989
rs2276948	4	54851157	A	G	19	245	792	5	0,1033	0,1212	0,394
rs13147194	4	54851891	T	G	792	248	21	0	-0,1163	0,1281	0,3639
rs55784333	4	54854148	C	G	798	245	18	0	-0,0805	0,1307	0,538
rs3733540	4	54856011	T	C	792	248	21	0	-0,1163	0,1281	0,3639
rs4864878	4	54868472	T	C	149	488	424	0	0,0743	0,0909	0,4138
rs6858442	4	54868690	A	G	192	497	370	2	0,0849	0,0875	0,3323
rs28889275	4	54870830	A	G	149	488	422	2	0,0661	0,0901	0,463
rs17084148	4	54891377	T	C	146	487	427	1	0,0782	0,0907	0,3886
rs73252935	4	54710879	T	C	874	180	7	0	0,004	0,155	0,9792
rs62297645	4	54732370	A	G	844	190	18	9	0,0601	0,1353	0,6571
rs6852007	4	54739176	A	G	11	193	856	1	0,0853	0,1436	0,5524

rs13135841	4	54740496	T	C	174	515	371	1	-0,281	0,0895	0,0017
rs13145280	4	54751622	T	C	75	382	602	2	0,1935	0,0984	0,0494
rs11133311	4	54762121	T	C	158	527	373	3	-0,3007	0,0904	0,0009
rs28622224	4	54782850	T	C	74	404	582	1	0,1039	0,0995	0,2966
rs4864857	4	54784571	T	C	680	326	36	19	-0,0596	0,1083	0,5819
rs7698425	4	54785413	T	C	37	328	694	2	0,0793	0,1109	0,4749
rs1800810	4	54788788	C	G	692	327	37	5	-0,1224	0,1117	0,2733
rs1800813	4	54789224	A	G	34	330	696	1	0,0604	0,1132	0,594
rs7689569	4	54791155	A	G	35	326	698	2	0,0777	0,1118	0,4871
rs7679903	4	54792130	T	C	696	328	37	0	-0,0953	0,1132	0,3999
rs6554163	4	54797316	A	T	36	325	696	4	0,0678	0,1093	0,5347
rs67432867	4	54799211	A	T	694	329	37	1	-0,096	0,1128	0,3947
rs73252942	4	54799361	T	C	692	331	35	3	-0,0732	0,113	0,5172
rs4864864	4	54827082	T	C	17	209	834	1	0,0719	0,1346	0,5931
rs41279519	4	54828988	C	G	5	154	899	3	0,2559	0,1547	0,0985
rs869978	4	54834773	T	C	39	316	706	0	-0,0365	0,1128	0,7466
rs73252950	4	54836257	T	C	9	181	843	28	-0,0803	0,1026	0,4343
rs67388297	4	54837448	C	G	859	190	12	0	-0,0103	0,1459	0,9436
rs28698464	4	54837867	A	G	842	207	12	0	-0,1139	0,1423	0,4237
rs1316926	4	54838043	A	G	257	516	242	46	0,1026	0,0785	0,1915
rs28374326	4	54838078	T	C	5	192	859	5	-0,046	0,1397	0,7417
rs2412556	4	54840015	A	G	784	253	21	3	-0,1256	0,1263	0,3203
rs2162136	4	54756155	T	G	43	355	662	1	0,097	0,1088	0,3729
rs6832597	4	54770994	A	C	41	351	668	1	0,0933	0,1096	0,395
rs7681399	4	54785643	T	G	695	328	38	0	-0,1071	0,1127	0,3422
rs1800812	4	54789386	T	G	37	328	696	0	0,0953	0,1132	0,3999
rs4864862	4	54795246	A	G	37	328	696	0	0,0953	0,1132	0,3999
rs4864863	4	54795588	A	G	696	328	37	0	-0,0953	0,1132	0,3999
rs2229307	4	54824835	T	C	834	210	17	0	-0,1102	0,1367	0,4203
rs2307049	4	54824911	A	G	18	209	834	0	0,1195	0,1359	0,3796
rs7686588	4	54829385	A	G	832	210	18	1	-0,1202	0,1353	0,3746
rs12644709	4	54830337	A	G	834	208	17	2	-0,0955	0,1361	0,4832
rs1547904	4	54841146	T	C	20	249	792	0	0,0755	0,1287	0,5578
rs2412557	4	54844214	A	C	785	254	22	0	-0,0957	0,1267	0,45
rs4864872	4	54847041	T	G	20	256	782	3	0,1116	0,1227	0,3633
rs10020847	4	54847891	T	C	19	250	789	3	0,0256	0,124	0,8362
rs10021728	4	54848866	T	C	19	250	792	0	0,0673	0,1294	0,6032
rs11733839	4	54849284	C	G	22	253	786	0	0,1029	0,1268	0,4173

Appendix Table S1: SNPs in preterm infants with and without BPD Genetic association results of PDGF-R□ related SNPs with BPD. SNPs with p-values≤0.001 are marked in red. SNPS with p-value≤0.05 are marked in yellow. Position information is referring to genome build hg18.

Appendix Table S2.

Pathway	Nominally associated genes / all pathway genes	Nominally associated genes	Best p-values of nominally associated genes	Reference eQTL database
**JAK / STAT Cascade**	6 / 10	*EPS8, GRB2, NUP62, PPP2CA, STAT1, STAT3*	0.0013-0.044	dixon, fehrmann, kirsten_2014, seeQTL, zeller
**MAPKKK Cascade**	7 / 14	*DUSP1, DUSP6, EGF, MAP2K1, MAPK9, PPP2CA, PRKCA*	0.00015-0.044	dixon, fehrmann, kirsten_2014, seeQTL, zeller
**Apoptosis**	8 / 22	*AKT1, LTA, NFKB1, NUP62, PPP2CA, PRKCA, RASA1, STAT1*	0.00015-0.044	dixon, fehrmann, kirsten_2014, seeQTL, zeller
**Cell Cycle**	5 / 19	*DUSP1, DUSP6, PPP2CA, PRKCA, STAT1*	0.00015-0.044	dixon, fehrmann, kirsten_2014, seeQTL, zeller
**DNA Metabolism**	5 / 9	*ATF2, CREB1, EGF, NUP62, PPP2CA*	0.0013-0.044	dixon, kirsten_2014
**Lipid Metabolism**	3 / 9	*PIK3R1, PPP2CA, PRKCA*	0.00015-0.044	dixon, kirsten_2014, seeQTL, zeller
**Protein Metabolism**	14 / 39	*ACTR2, AKT1, ATF2, CREB1, DUSP1, DUSP6, GSK3B, IKBKB, MAP2K1, MAP3K2, MAPK9, PPP2CA, PRKCA, RPS6KA5*	0.00015-0.044	ding, dixon, fehrmann, kirsten_2014, seeQTL, zeller
**Actin and Calcium Ion Homeostasis**	6 / 15	*ACTR2, EGF, PRKCA, RASA1, STAT1, STAT3*	0.00015-0.042	ding, dixon, kirsten_2014, seeQTL, zeller

Appendix Table S2: Analysis of SNPs in PDGF-related pathways PDGF-related pathways with overrepresentation of nominally BPD-associated cis-eQTL SNPs.

Appendix Table S3.

A

**Patients**	n=9
**BPD grades**	BPD grade 0 n=2
	BPD grade 1/2/3 n=7
**Gestational age (weeks)**	26.5 +-2.4
**Birth weight (grams)**	858.9 +-234
**Males/Females**	6/3

B

**Patients**	n=13
**BPD grades**	BPD grade 0/1 n=9
	BPD grade 2/3 n=4
**Gestational age (weeks)**	26.2±1
**Birth weight (gms)**	774.6±136
**Mechanical Ventilation (days)**	56.3±14
**Males/Females**	n=5/n=8
**Early onset infection**	n=6

C

**Patients**	n=6
**BPD grades**	BPD grade 0/1 n=2
	BPD grade 2/3 n=4
**Gestational age (weeks)**	25 ± 1
**Birth weight (gms)**	563 ± 98
**Mechanical Ventilation (days)**	74 ± 23
**Males/Females**	n=4/n=2
**Days of culture (days)**	3-14
**Congenital infection**	n=2

D

**Patients**	n=8
**BPD grades**	BPD grade 0 n=1
	BPD grade 1/2/3 n=7
**Gestational age (weeks)**	27.3±2
**Age of death (days)**	37.75
**Died of respiratory failure**	n=6
**Males/Females**	n=5/n=3

BPD grade **1=** O2 requirement at day 28 of life, **2=** O2 requirement of <30% at 36 weeks, **3=** O2 requirement of >30% at 36 weeks and ventilator support.

Appendix Table S3. Characteristics of patients from which the samples for (A) protein data corresponding to SNP analysis (B) gene expression data corresponding to SNP analysis were derived and (C) isolation of fibroblasts.

APPENDIX REFERENCES

Bolstad BM, Irizarry RA, Astrand M, Speed TP (2003) A comparison of normalization methods for high density oligonucleotide array data based on variance and bias. Bioinformatics 19**:** 185-193

Devlin B, Roeder K (1999) Genomic control for association studies. Biometrics 55**:** 997-1004

Ding J, Gudjonsson JE, Liang L, Stuart PE, Li Y, Chen W, Weichenthal M, Ellinghaus E, Franke A, Cookson W et al (2010) Gene expression in skin and lymphoblastoid cells: Refined statistical method reveals extensive overlap in cis-eQTL signals. American journal of human genetics 87**:** 779-789

Dixon AL, Liang L, Moffatt MF, Chen W, Heath S, Wong KC, Taylor J, Burnett E, Gut I, Farrall M et al (2007) A genome-wide association study of global gene expression. Nature genetics 39**:** 1202-1207

Ewing B, Hillier L, Wendl MC, Green P (1998) Base-calling of automated sequencer traces using phred. I. Accuracy assessment. Genome research 8**:** 175-185

Fehrmann RS JR, Veldink JH, Westra HJ, Arends D, Bonder MJ, Fu J, Deelen P, Groen HJ, Smolonska A, Weersma RK, Hofstra RM, Buurman WA, Rensen S, Wolfs MG, Platteel M, Zhernakova A, Elbers CC, Festen EM, Trynka G, Hofker MH, Saris CG, Ophoff RA, van den Berg LH, van Heel DA, Wijmenga C, Te Meerman GJ, Franke L (2011) Trans-eqtls reveal that independent genetic variants associated with a complex phenotype converge on intermediate genes, with a major role for the hla. . PLoS genetics 7

Jobe AH (2011) The new bronchopulmonary dysplasia. Current opinion in pediatrics 23**:** 167-172

Kim KY, Kim BJ, Yi GS (2004) Reuse of imputed data in microarray analysis increases imputation efficiency. BMC bioinformatics 5**:** 160

Rohloff JC, Gelinas AD, Jarvis TC, Ochsner UA, Schneider DJ, Gold L, Janjic N (2014) Nucleic Acid Ligands With Protein-like Side Chains: Modified Aptamers and Their Use as Diagnostic and Therapeutic Agents. Molecular therapy Nucleic acids 3**:** e201

Xia K, Shabalin AA, Huang S, Madar V, Zhou YH, Wang W, Zou F, Sun W, Sullivan PF, Wright FA (2012) seeQTL: a searchable database for human eQTLs. Bioinformatics 28**:** 451-452

Zeller T, Wild P, Szymczak S, Rotival M, Schillert A, Castagne R, Maouche S, Germain M, Lackner K, Rossmann H et al (2010) Genetics and beyond--the transcriptome of human monocytes and disease susceptibility. PloS one 5**:** e10693
