# Supplementary material for: Attenuated PDGF signaling drives alveolar and microvascular defects in neonatal chronic lung disease
Source: EMBO Mol Med. 2017 Sep 18;9(11):1504–20. doi: 10.15252/emmm.201607308 (PMC5666314; doi:10.15252/emmm.201607308)
Supplement: Supplementary file 2 — Expanded View Figures PDF [file EMMM-9-1504-s002.pdf]

Expanded View Figures

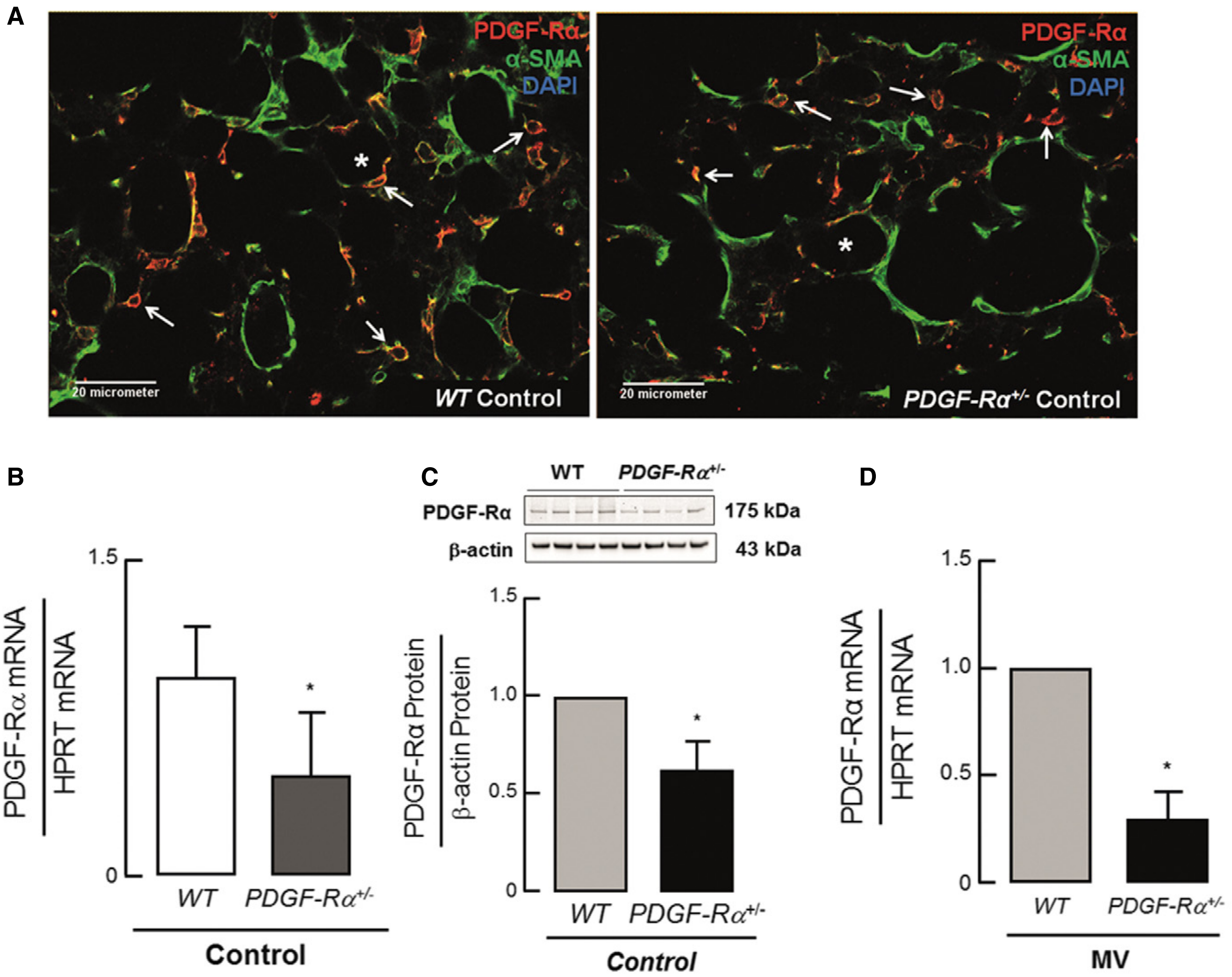

Figure EV1. Reduced PDGF-Rα abundance in PDGF-Rα haploinsufficient mice.

A Double staining for PDGF-Rα (red) and α-SMA (green) showed a co-localization of these two proteins as well as a reduced number of double-positive cells (white arrows) in 5–8-day-old PDGF-Rα$^{+/-}$ mice (right panel) when compared to PDGF-Rα$^{+/+}$ (WT) littermates (left panel). * represents alveolar air space.

B, C (B) Quantitative RT–PCR and (C) immunoblot analysis showing reduced PDGF-Rα protein and mRNA expression in lungs of PDGF-Rα$^{+/-}$ newborn mice when compared to WT littermates (n = 4/group).

D Reduced PDGF-Rα transcript in newborn PDGF-Rα$^{+/-}$ mice upon MV-O_2 for 8 h when compared to WT littermates (n = 3 mice/group).

Data information: In (B–D), the data are presented as mean ± SD. *$P < 0.05$. Statistical test in (B–D) is two-tailed unpaired Student's t-test or Mann–Whitney test.

 © 2017 Helmholtz Zentrum München

Figure EV2. Effect of TGF-β in combination with mechanical stretch on PDGF-Rα signaling and functional properties of fibroblasts.

A No change in migration of myofibroblasts (MFBs) from newborn WT mice with mechanical stretch (n = 5 mice/group).

B–D Analysis of proliferation (Cell Titer Glo) exhibited no change upon TGF-β (B) or stretch (C) in mouse myofibroblasts (MFBs) from WT mice, while an increase in proliferation was observed when mouse myofibroblasts stretched in the presence of TGF-β were subjected to an additional dose of TGF-β (D) (n = 9 mice/group).

E, F Immunoblot analysis showing increased proliferation markers like PCNA (E) levels with mechanical stretch and PI3K (F) upon additional TGF-β incubation on stretched myofibroblasts (MFBs) from newborn WT mice (n = 6 mice/group).

Data information: Values here are normalized to respective controls. Data are presented as mean ± SD. Statistical analysis in (A–C, E) is two-tailed unpaired Student's t-test and in (D, F) is ordinary one-way ANOVA with Bonferroni's correction. **$P < 0.01$, *$P < 0.05$ (P-value range = 0.0080–0.0232). C, un-stretched untreated control; S, stretched myofibroblasts (24 h); Th1, un-stretched myofibroblasts subjected to 5 ng/ml TGF-β (24 h); S+Th1, myofibroblasts stretched in parallel to TGF-β application (5 ng/ml) (24 h); S+Th2, myofibroblasts stretched for 24 h followed by incubation with TGF-β (5 ng/ml) for 24 h.

© 2017 Helmholtz Zentrum München

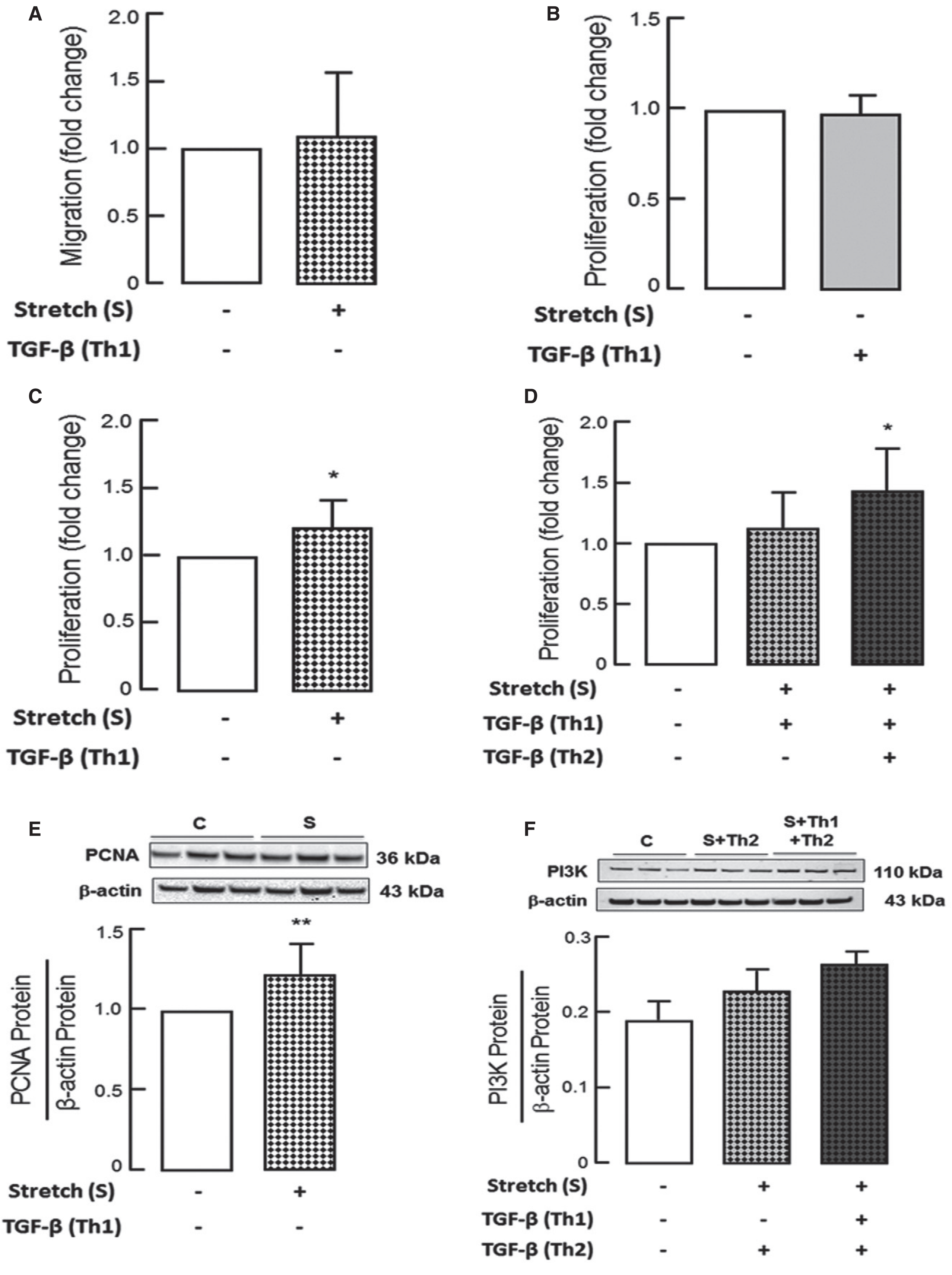

Figure EV2.

 © 2017 Helmholtz Zentrum München

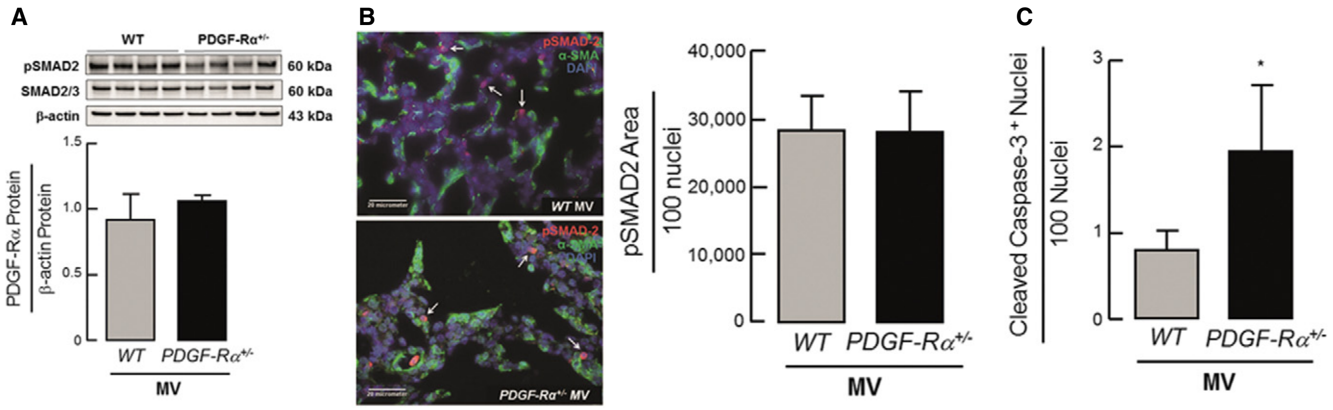

Figure EV3. Similar TGF-β activation with increased apoptosis in lungs of newborn PDGF-Rα haploinsufficient mice after MV-O_2 for 8 h.

A Immunoblot analysis showing similar pSMAD levels in whole lung homogenate of newborn WT as well as PDGF-Rα$^{+/-}$ mice (n = 3 mice/group).

B Representative immunofluorescence image showing similar pSMAD levels (red) in both newborn WT (upper panel) and PDGF-Rα$^{+/-}$ mice (lower panel). α-SMA is in green, and nucleus is stained with DAPI (blue). Quantification of the image showing pSMAD-2 area to 100 nuclei (n = 3–4 mice/group, 4 sections/mice, 10 images/section).

C Increased apoptosis quantified by cleaved caspase-3^{+} nuclei normalized to 100 nuclei in newborn PDGF-Rα$^{+/-}$ compared to WT mice (n = 4 mice/group).

Data information: In (A–C), data are presented as mean ± SD. Statistical test is two-tailed unpaired Student's t-test (*P = 0.0159).

© 2017 Helmholtz Zentrum München
