# Supplementary figures and images for: Attenuated PDGF signaling drives alveolar and microvascular defects in neonatal chronic lung disease
Source: EMBO Mol Med. 2017 Sep 18;9(11):1504–20. doi: 10.15252/emmm.201607308 (PMC5666314; doi:10.15252/emmm.201607308)

## Extended Version Figure 1C

PDGF-R $\alpha$ , 175KDa

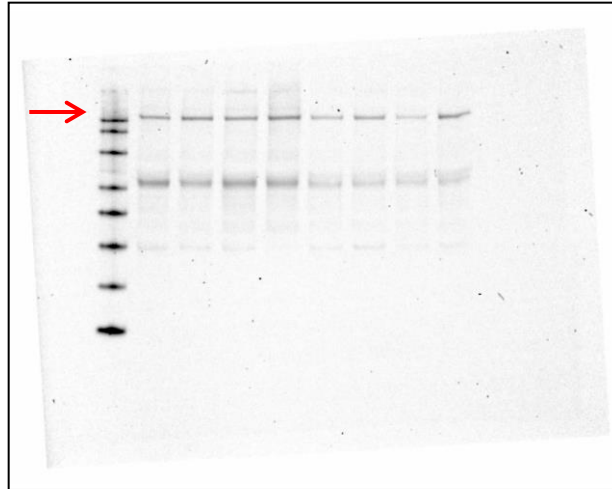

$\beta$ -actin, 43KDa

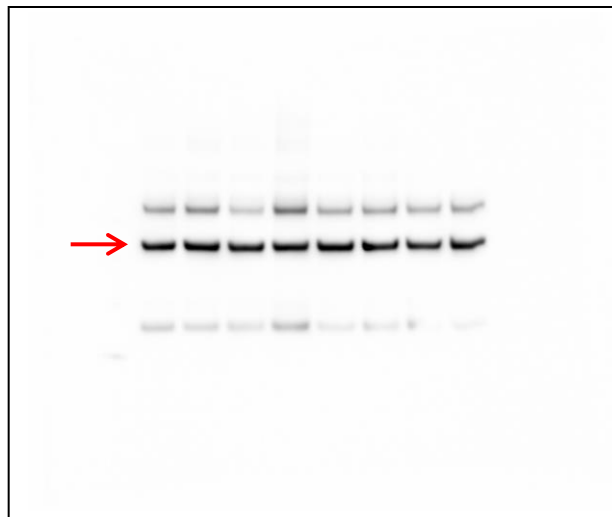

Supplement: Supplementary file 3 — Source Data for Expanded View [file EMMM-9-1504-s009.zip › EMM_07308_EV_source_data/EMM-2016-07308_uncut_blots_Fig._EV1.pdf]

Extended Version Figure 2E  
(Lanes 1-6)

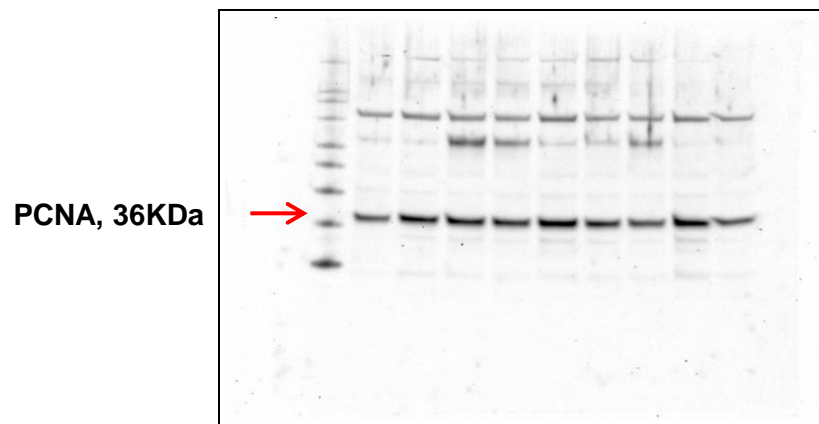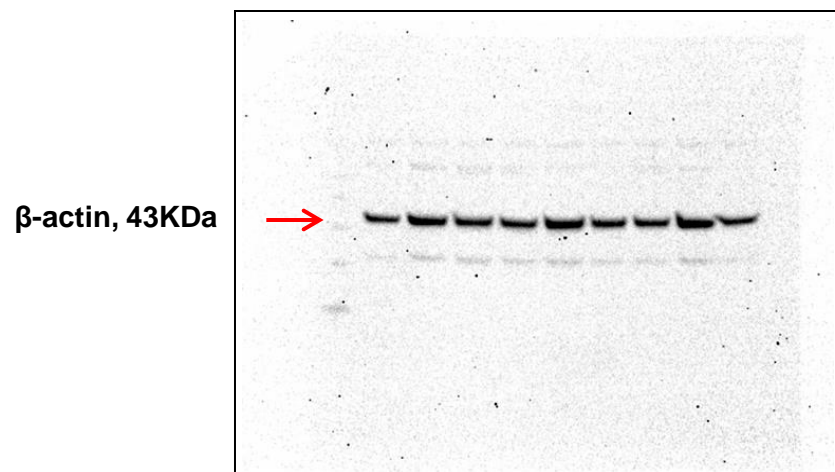

Extended Version Figure 2F

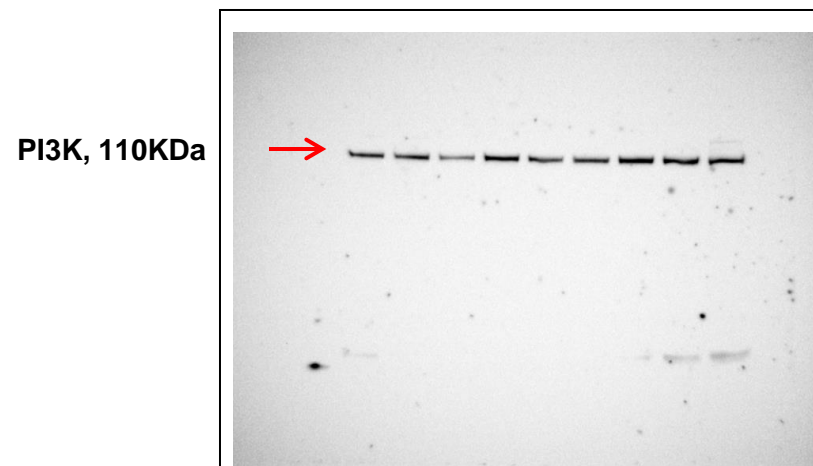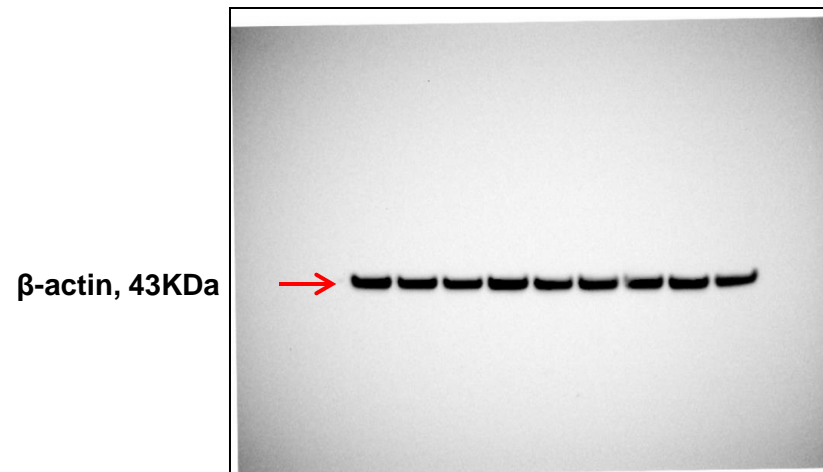

Supplement: Supplementary file 3 — Source Data for Expanded View [file EMMM-9-1504-s009.zip › EMM_07308_EV_source_data/EMM-2016-07308_uncut_blots_Fig._EV2.pdf]

# Extended Version Figure 3A

pSMAD 2, 60KDa

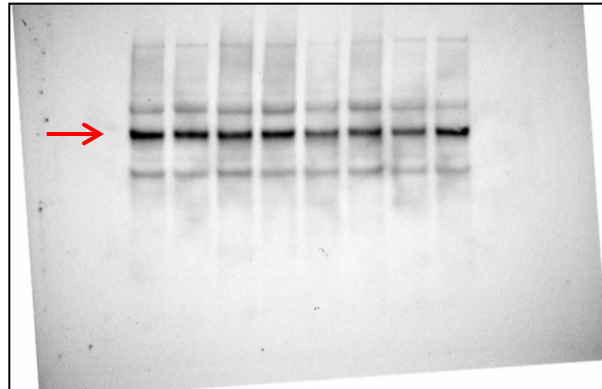

SMAD 2/3, 60KDa

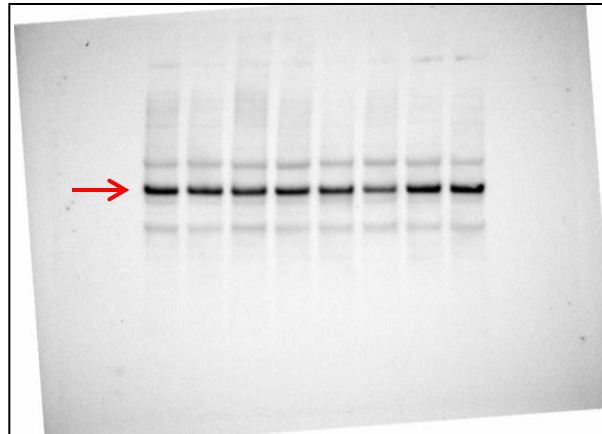

$\beta$ -actin, 43KDa

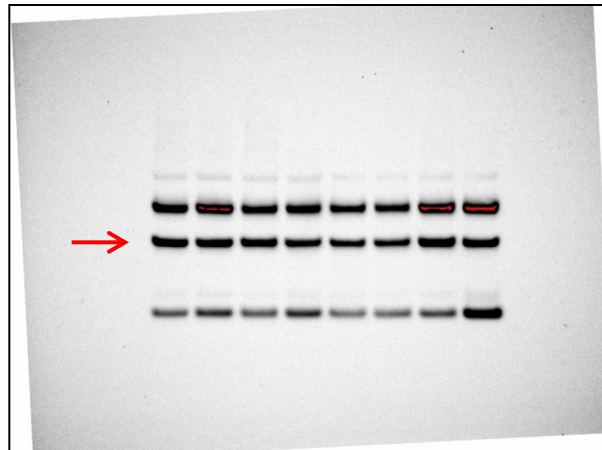

Supplement: Supplementary file 3 — Source Data for Expanded View [file EMMM-9-1504-s009.zip › EMM_07308_EV_source_data/EMM-2016-07308_uncut_blots_Fig._EV3.pdf]

Figure 1A

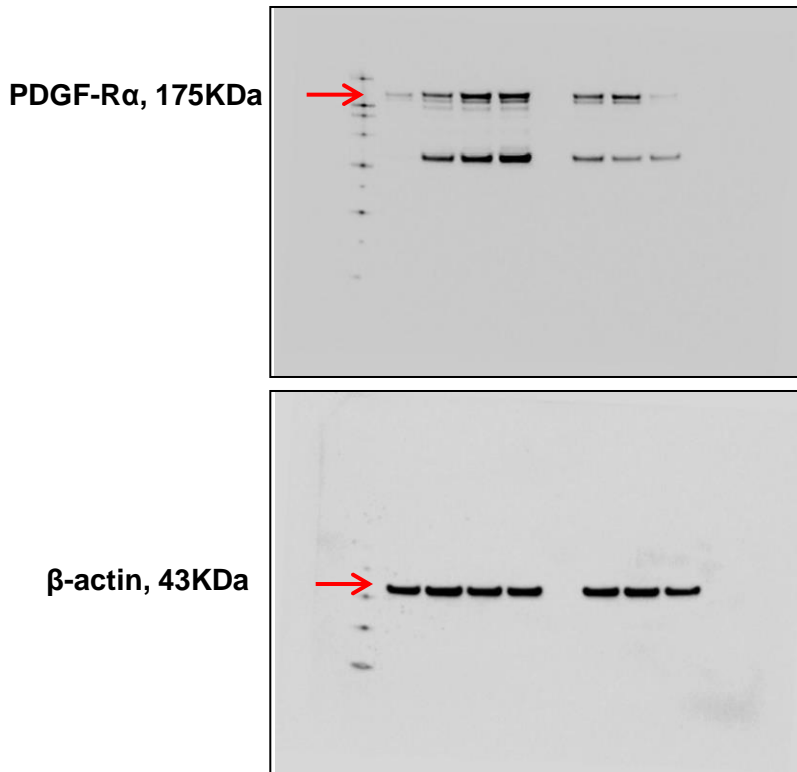

Figure 1F

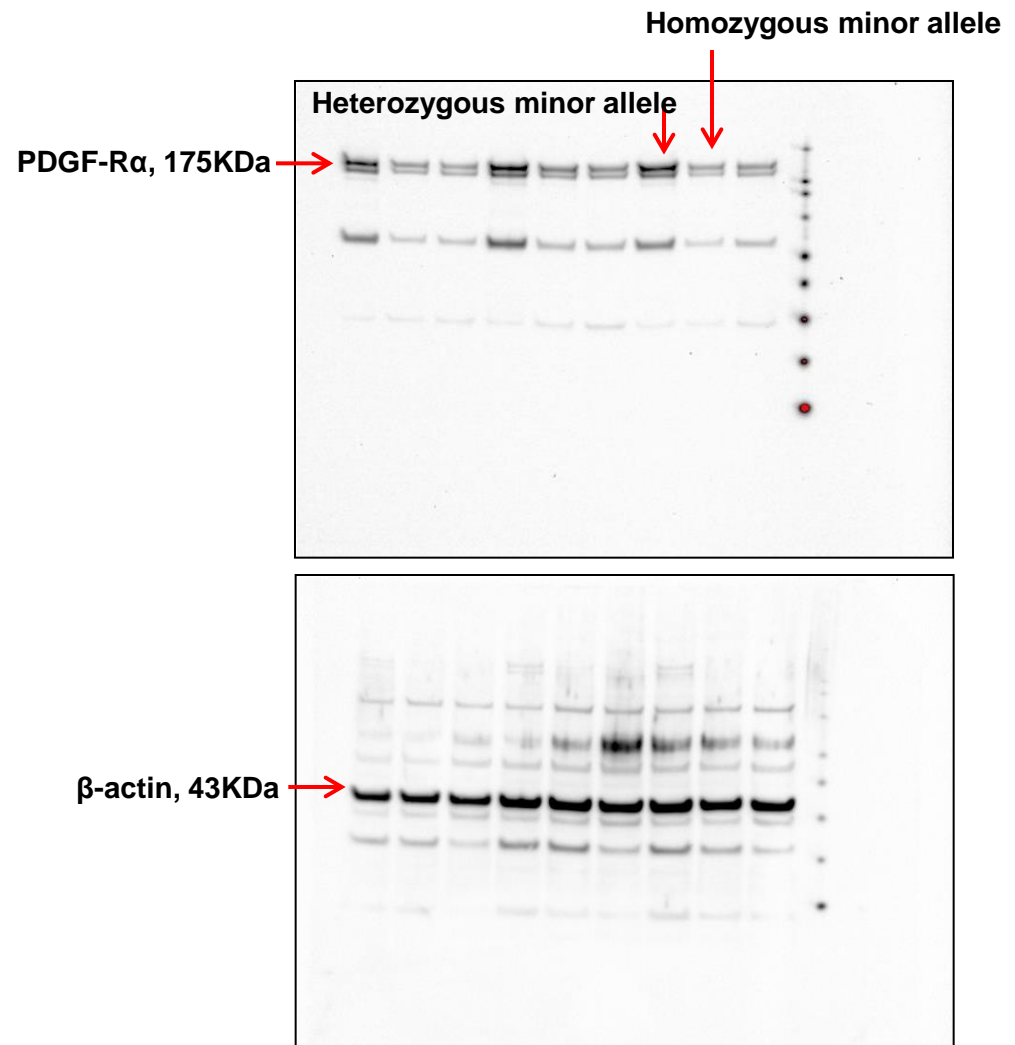

Supplement: Supplementary file 5 — Source Data for Figure 1 [file EMMM-9-1504-s003.pdf]

Figure 2G

PDGF-R $\alpha$ , 175KDa

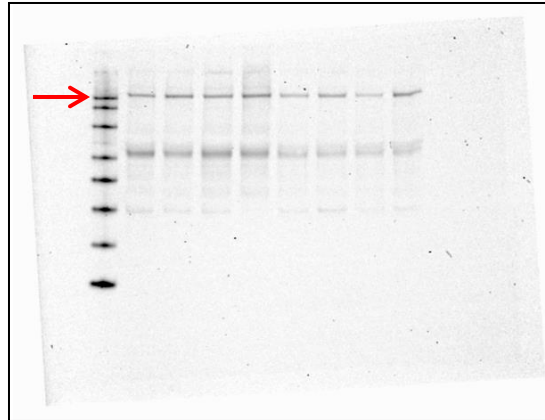

$\beta$ -actin, 43KDa

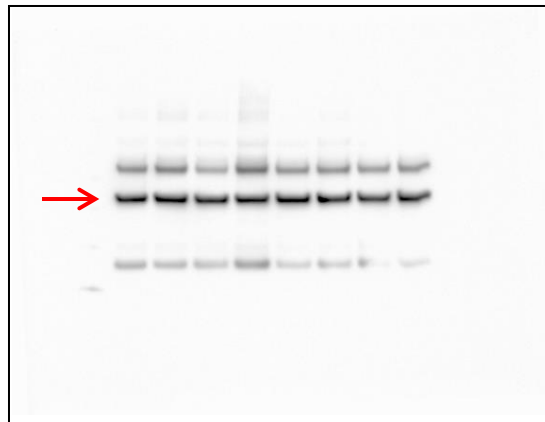

Figure 2H, I

JAK-2, 125KDa

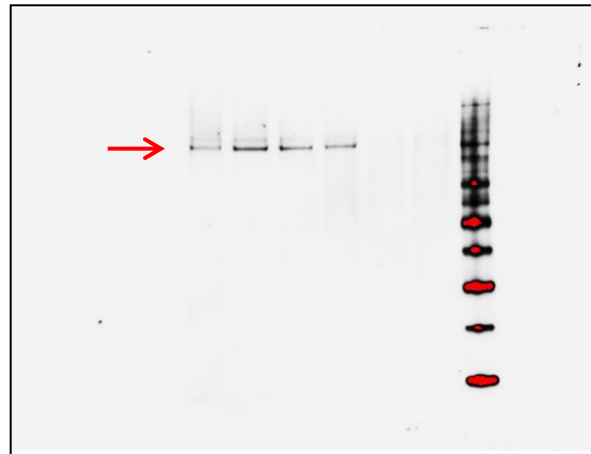

STAT-3, 86KDa

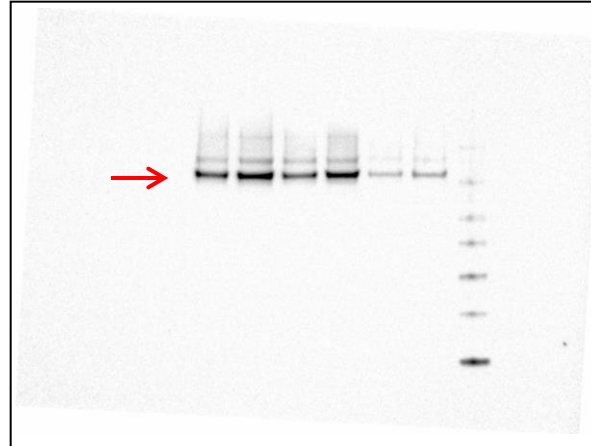

$\beta$ -actin, 43KDa

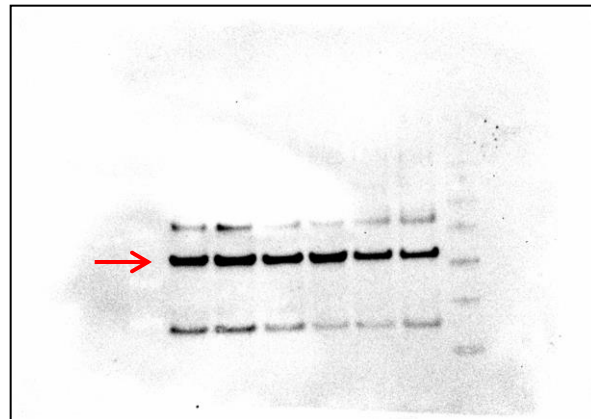

Supplement: Supplementary file 6 — Source Data for Figure 2 [file EMMM-9-1504-s004.pdf]

Figure 3B,C

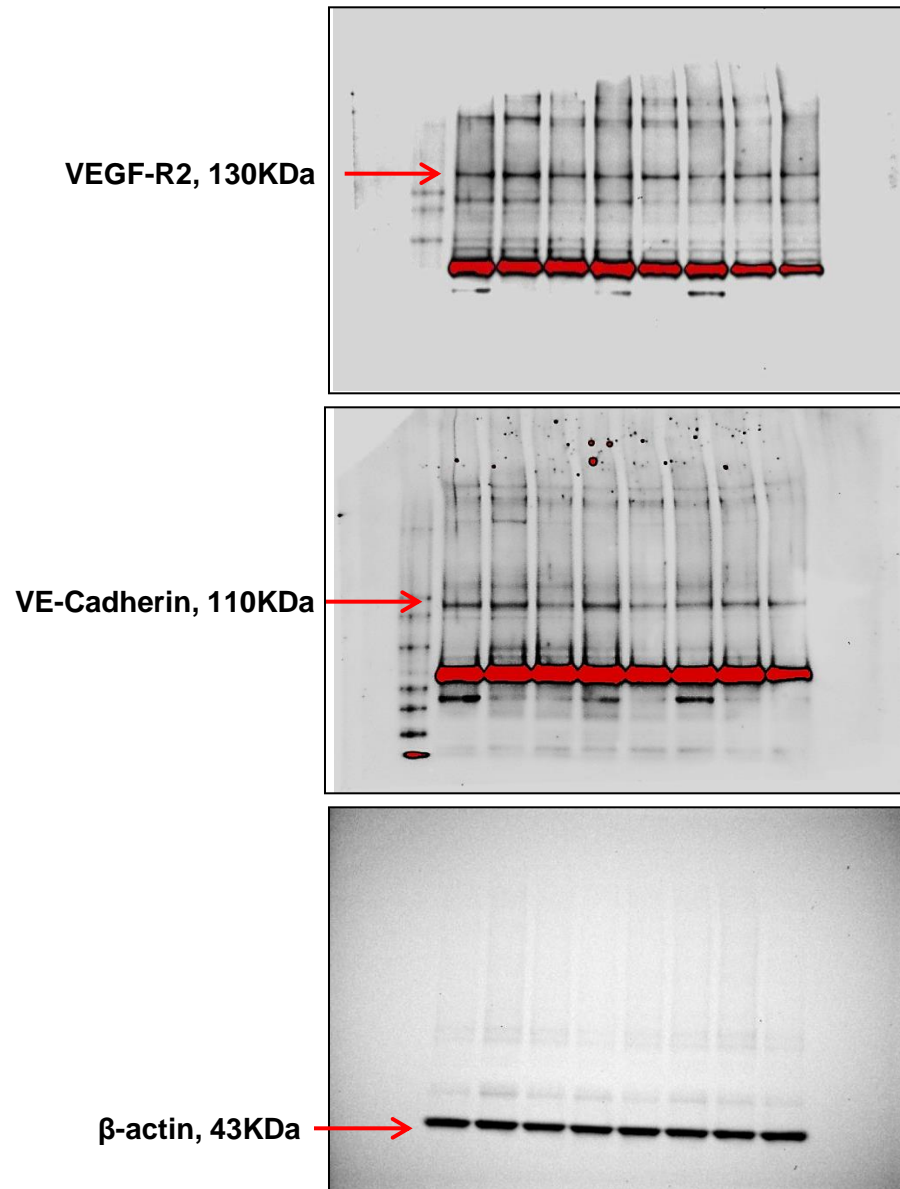

Figure 3D

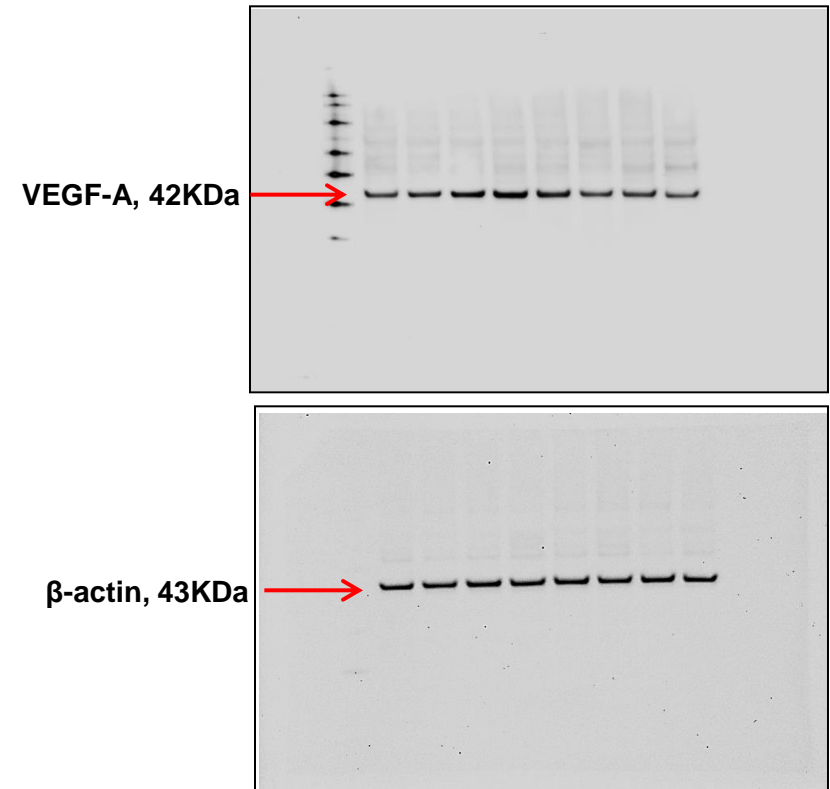

Figure 3H,I

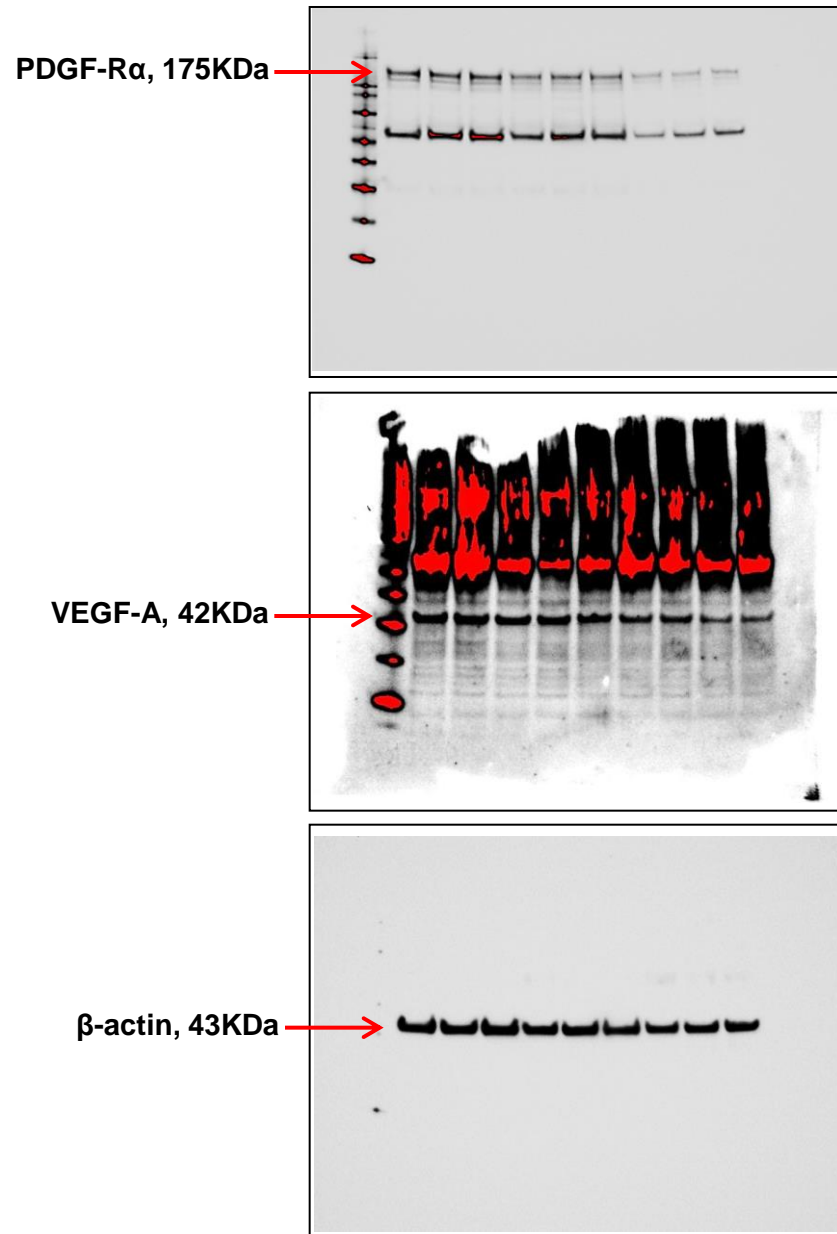

Figure 3J

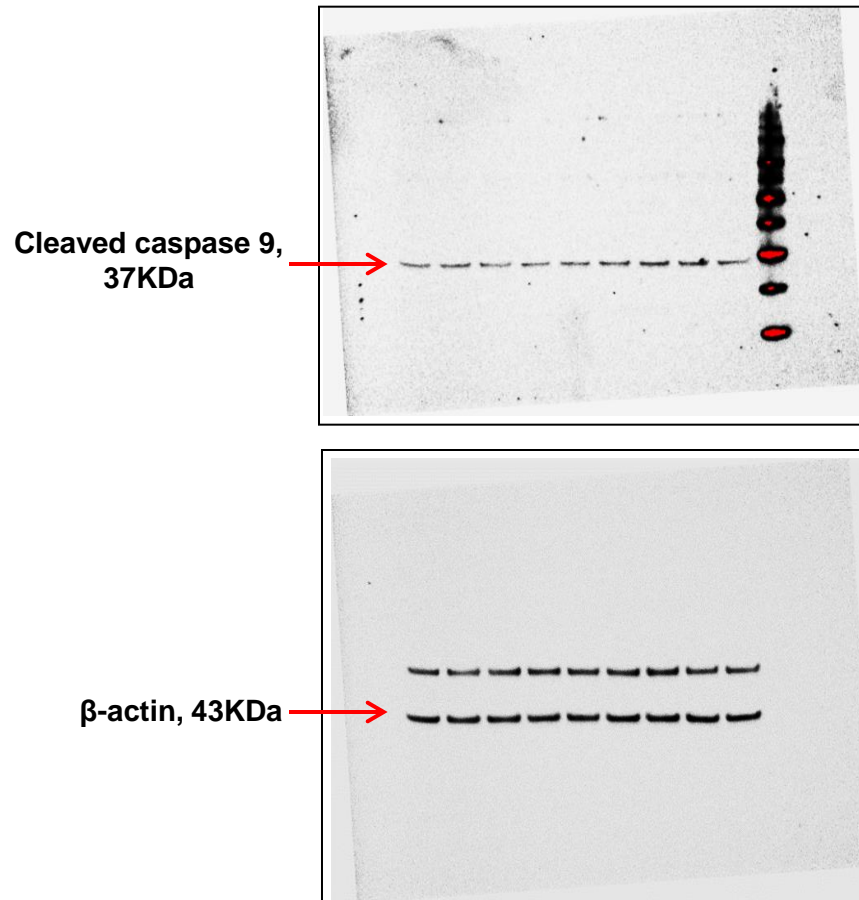

Figure 3K

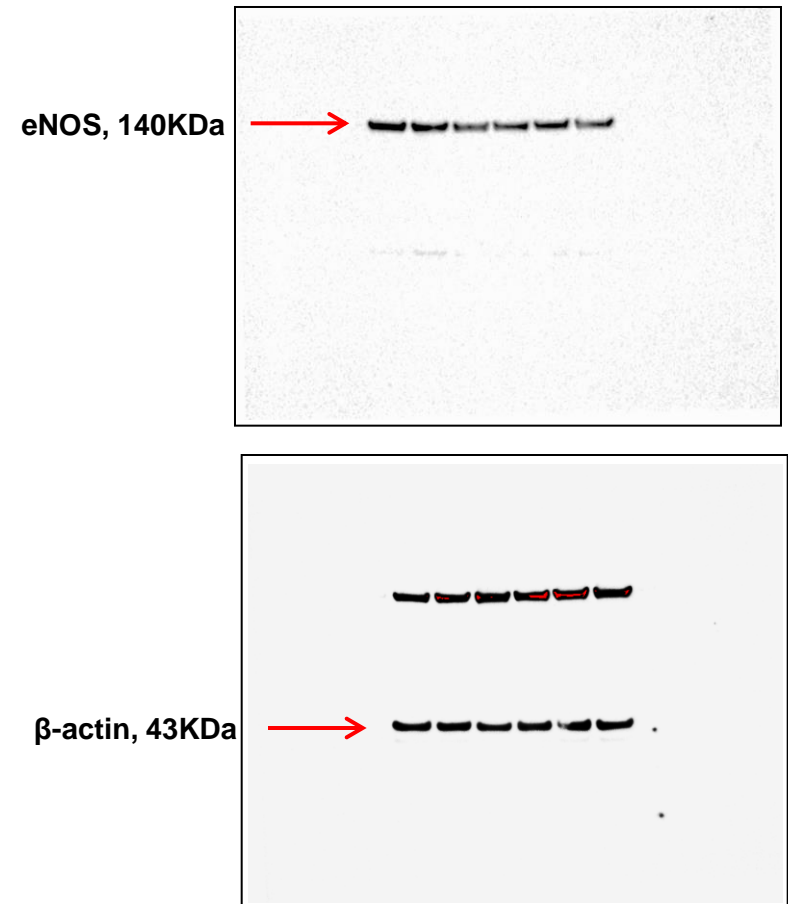

Supplement: Supplementary file 7 — Source Data for Figure 3 [file EMMM-9-1504-s005.pdf]

Figure 5E

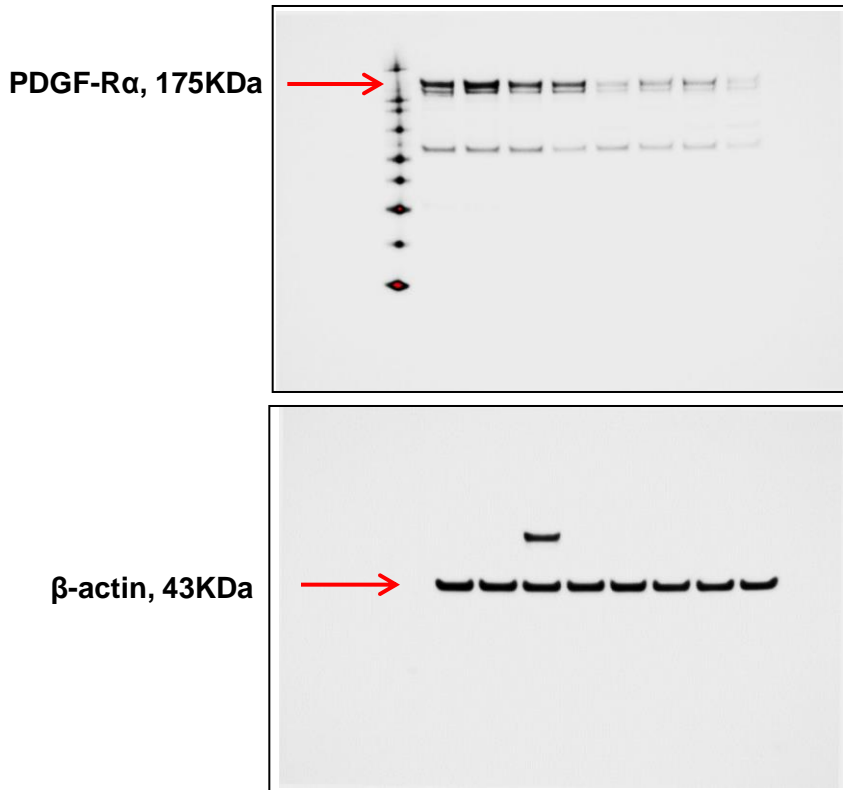

Figure 5F (Lanes 1-3 and 7-9)

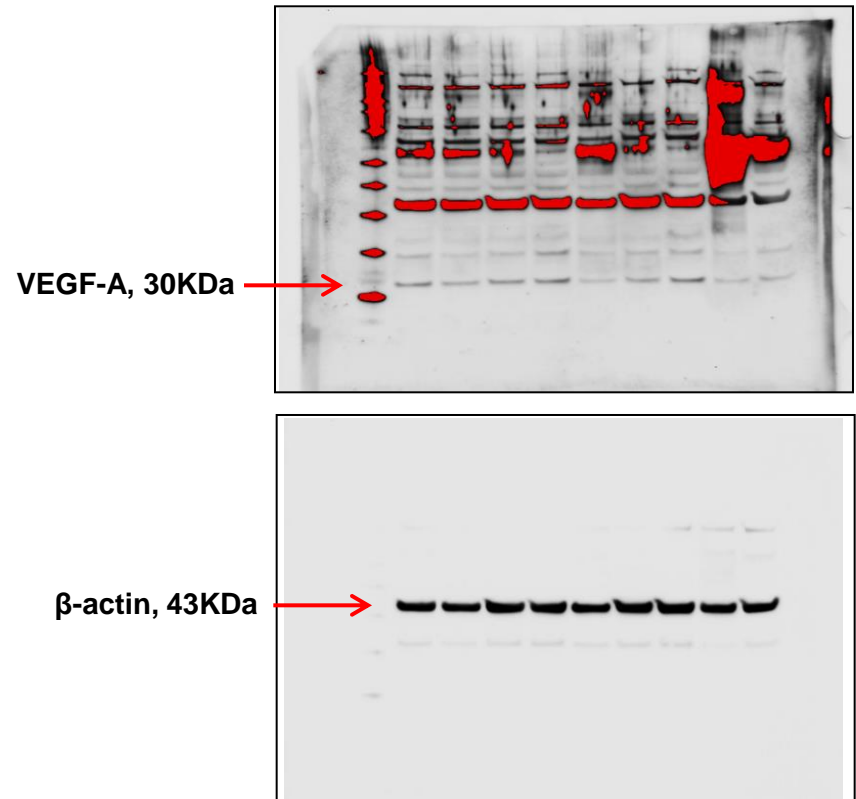

Figure 5G

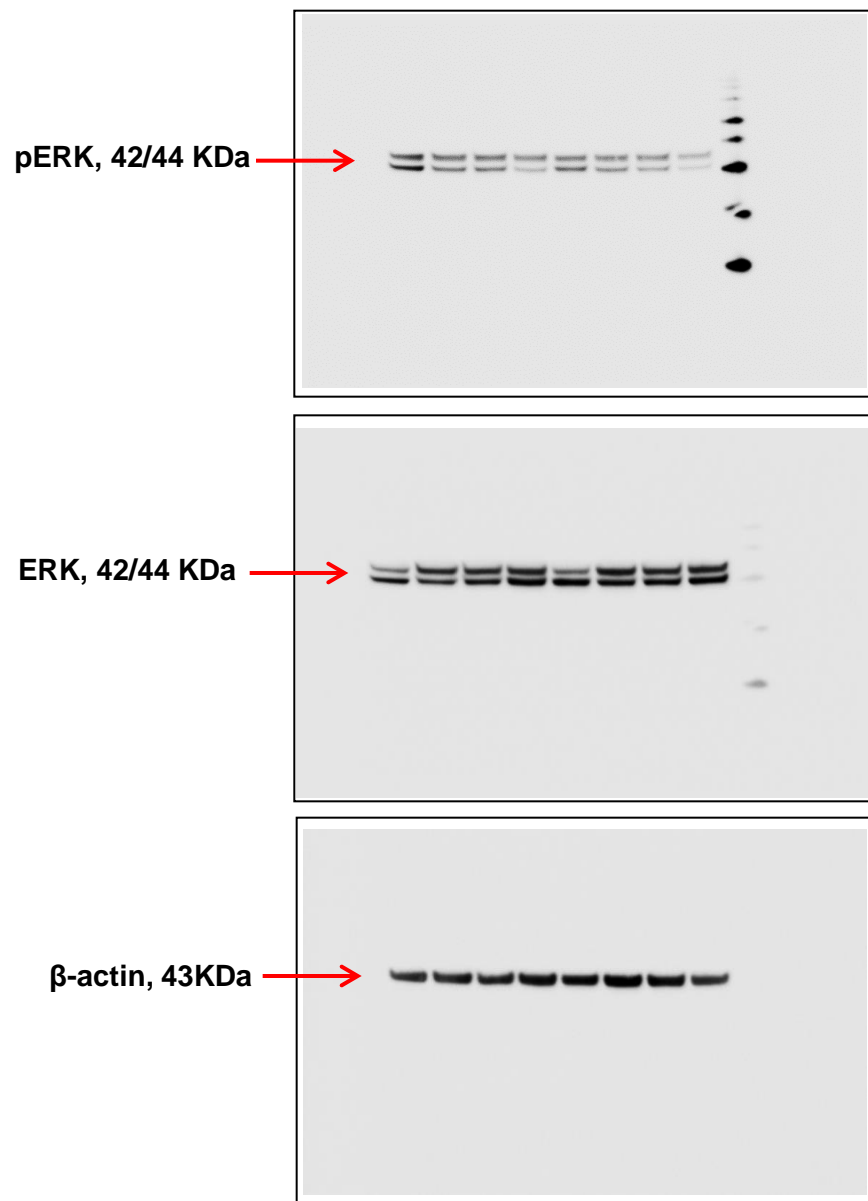

Figure 5I

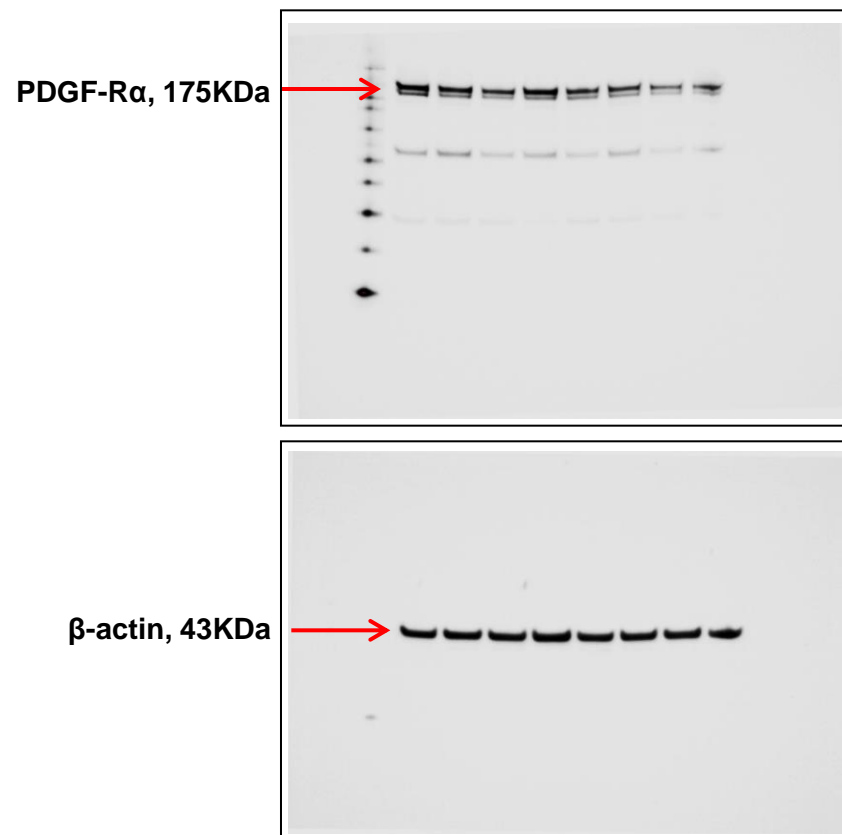

Supplement: Supplementary file 9 — Source Data for Figure 5 [file EMMM-9-1504-s007.pdf]
