# Supplementary material for: Attenuated PDGF signaling drives alveolar and microvascular defects in neonatal chronic lung disease
Source: EMBO Mol Med. 2017 Sep 18;9(11):1504–20. doi: 10.15252/emmm.201607308 (PMC5666314; doi:10.15252/emmm.201607308)

Figure 4D (Lanes 1-3 and 7-9)

PDGF-Rα, 175KDa

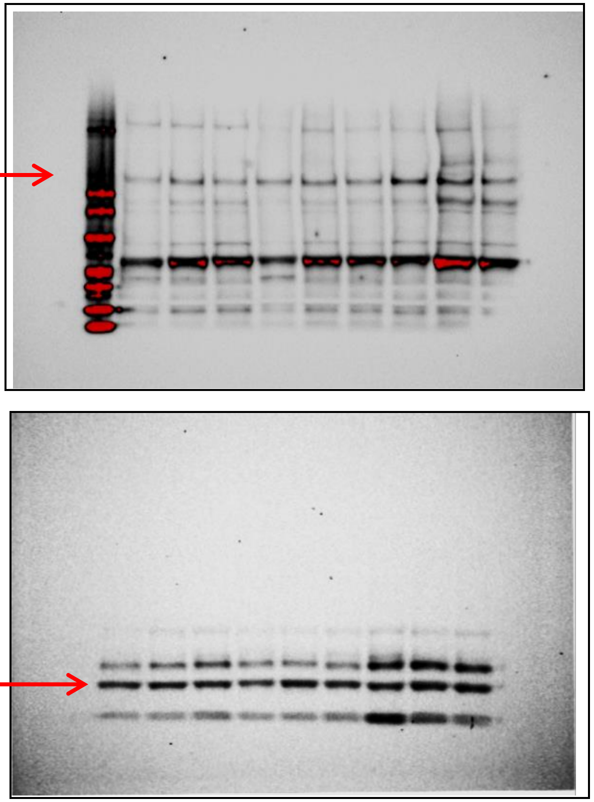

β-actin, 43KDa

Figure 4H (Lanes 1-3 and 7-9)

VE-Cadherin, 110KDa

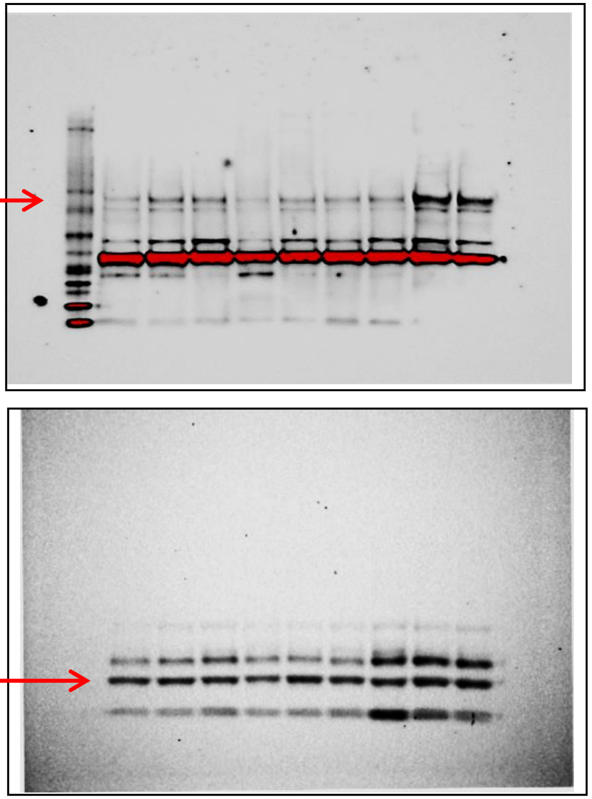

β-actin, 43KDa

Figure 4E and F

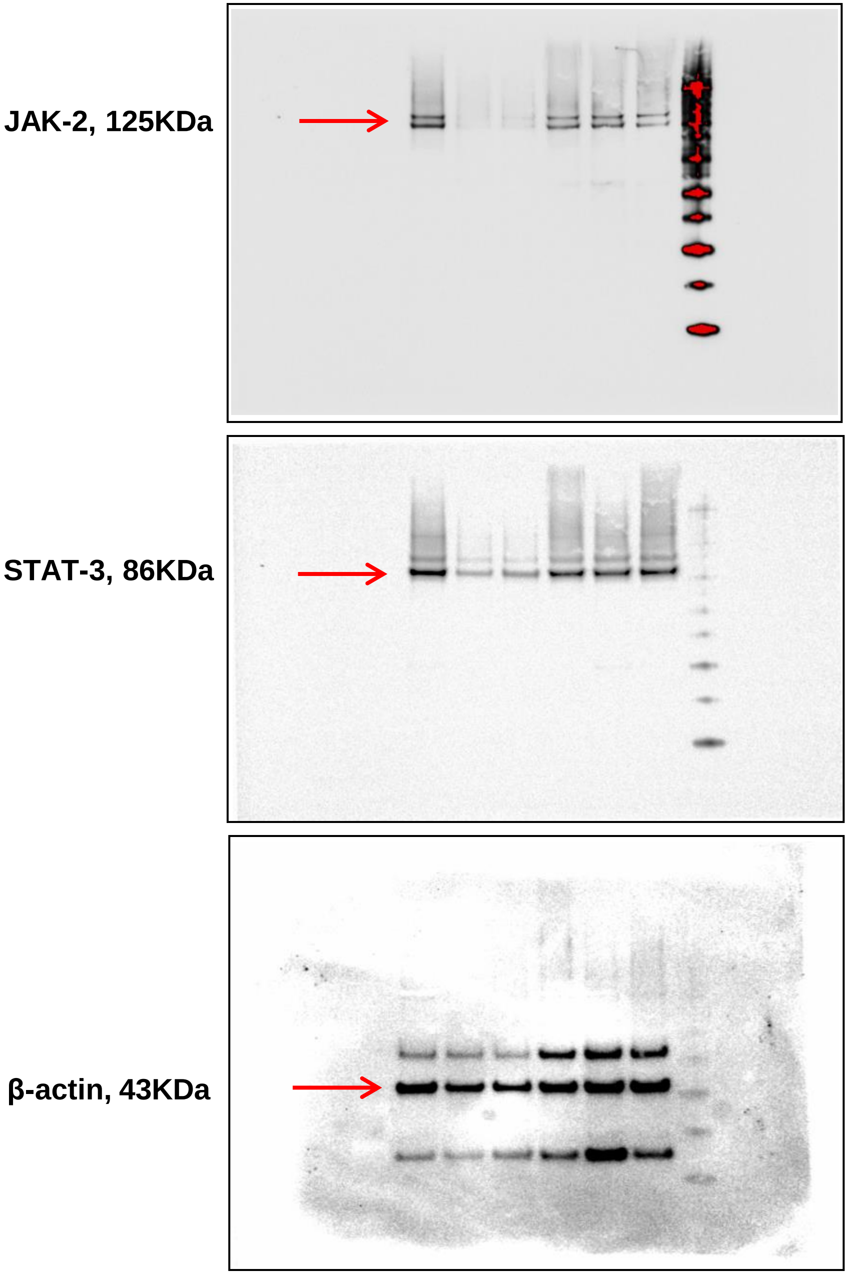

Figure 4G

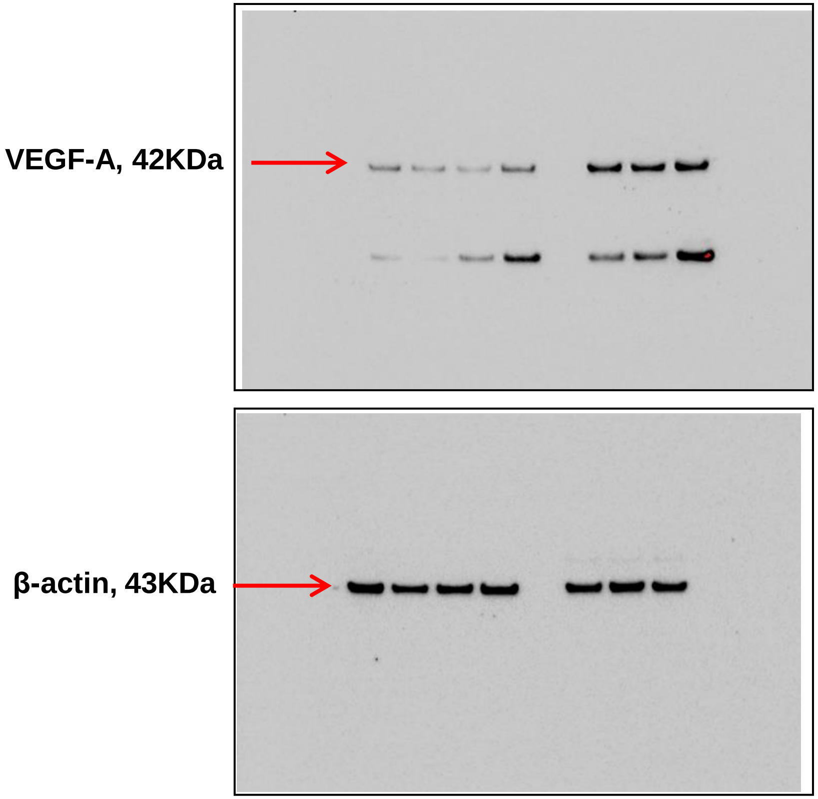

Figure 4I (Lanes 2-6)

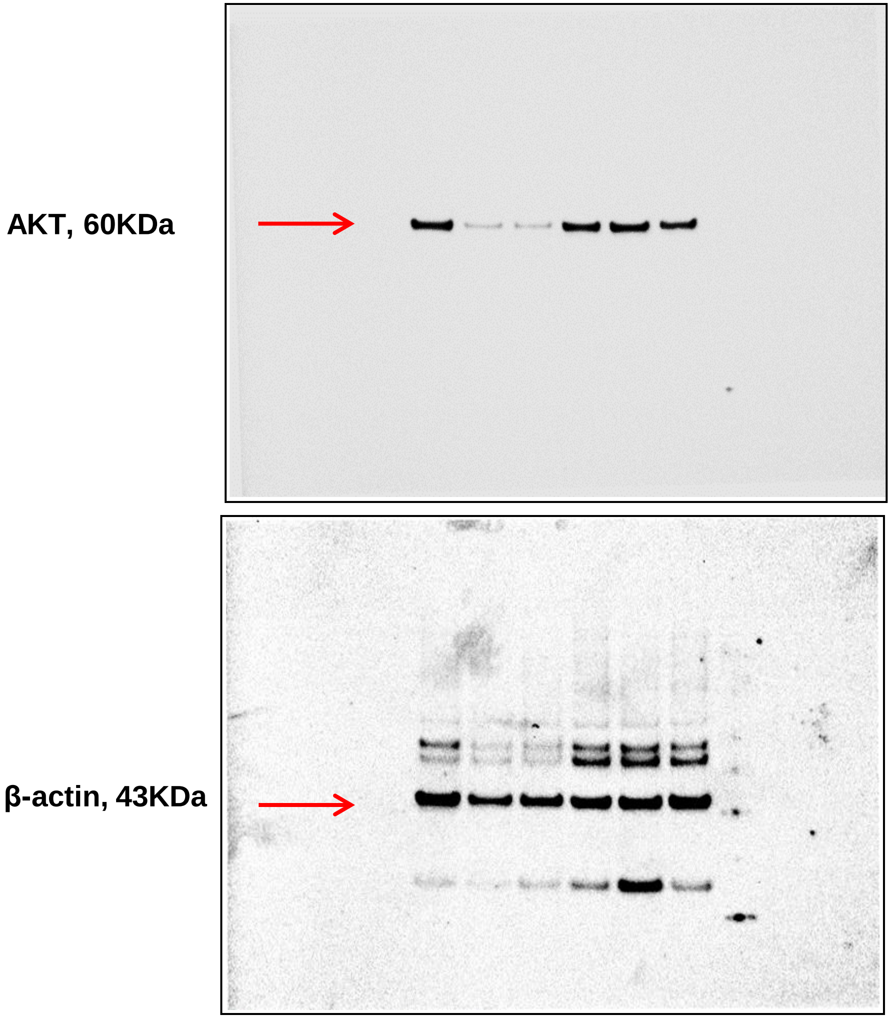

Supplement: Supplementary file 8 — Source Data for Figure 4 [file EMMM-9-1504-s006.pdf]
