# Supplementary material for: Attenuated PDGF signaling drives alveolar and microvascular defects in neonatal chronic lung disease
Source: EMBO Mol Med. 2017 Sep 18;9(11):1504–20. doi: 10.15252/emmm.201607308 (PMC5666314; doi:10.15252/emmm.201607308)

Figure 6A and E (Lanes 1-3 and 7-9)

PDGF-Rα, 175KDa

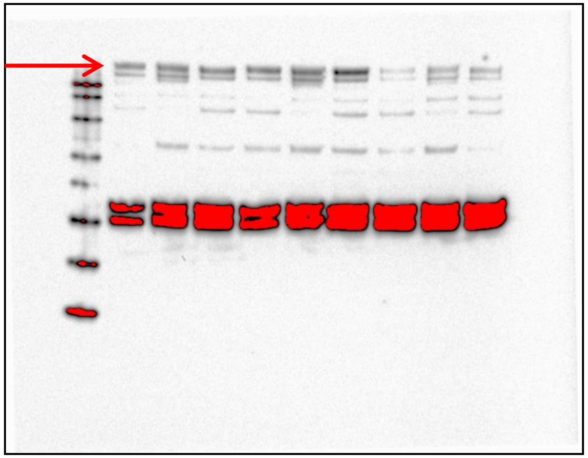

pERK, 42/44 KDa

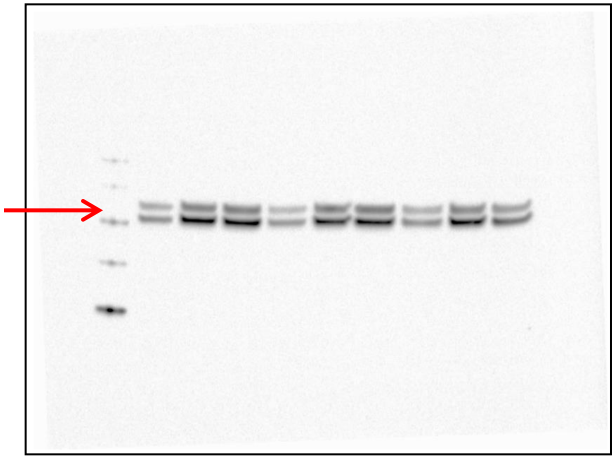

β-actin, 43KDa

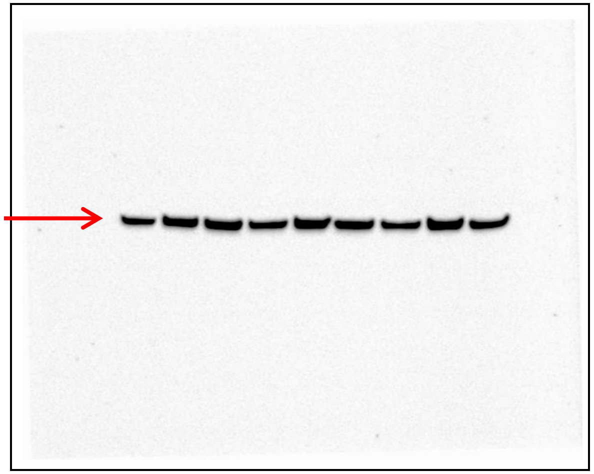

ERK, 42/44 KDa

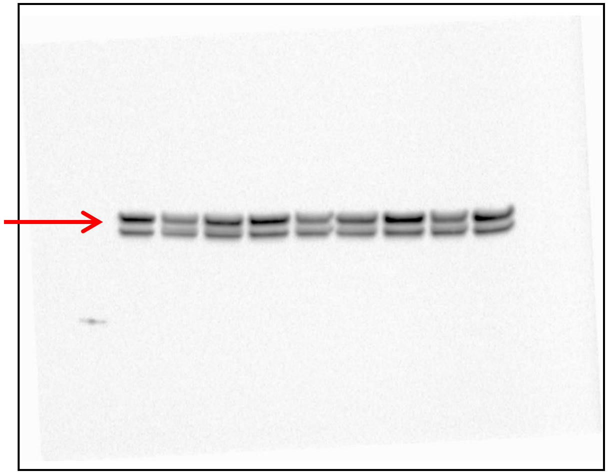

Figure 6B, C and D (Lanes 1-6)

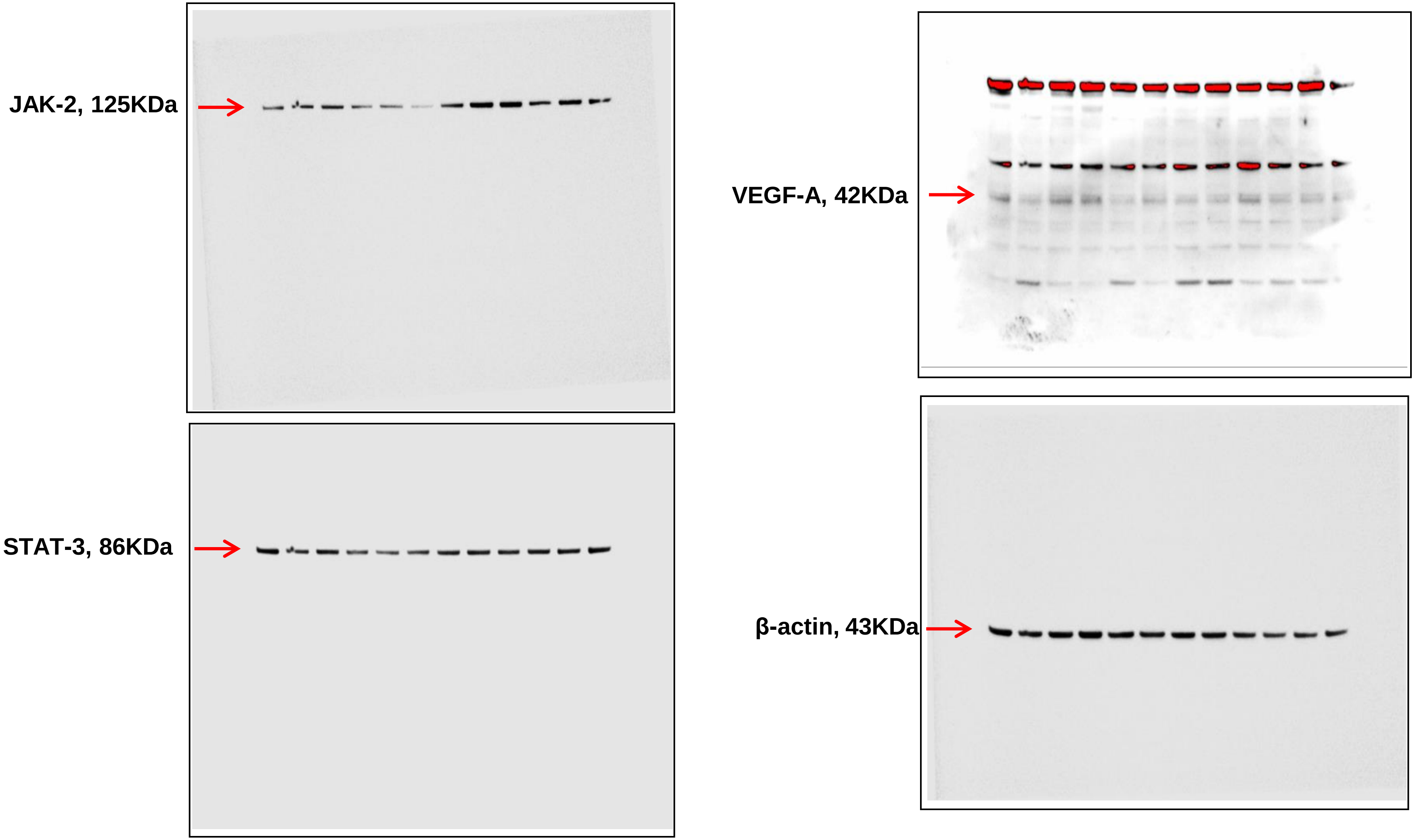

Figure 6G

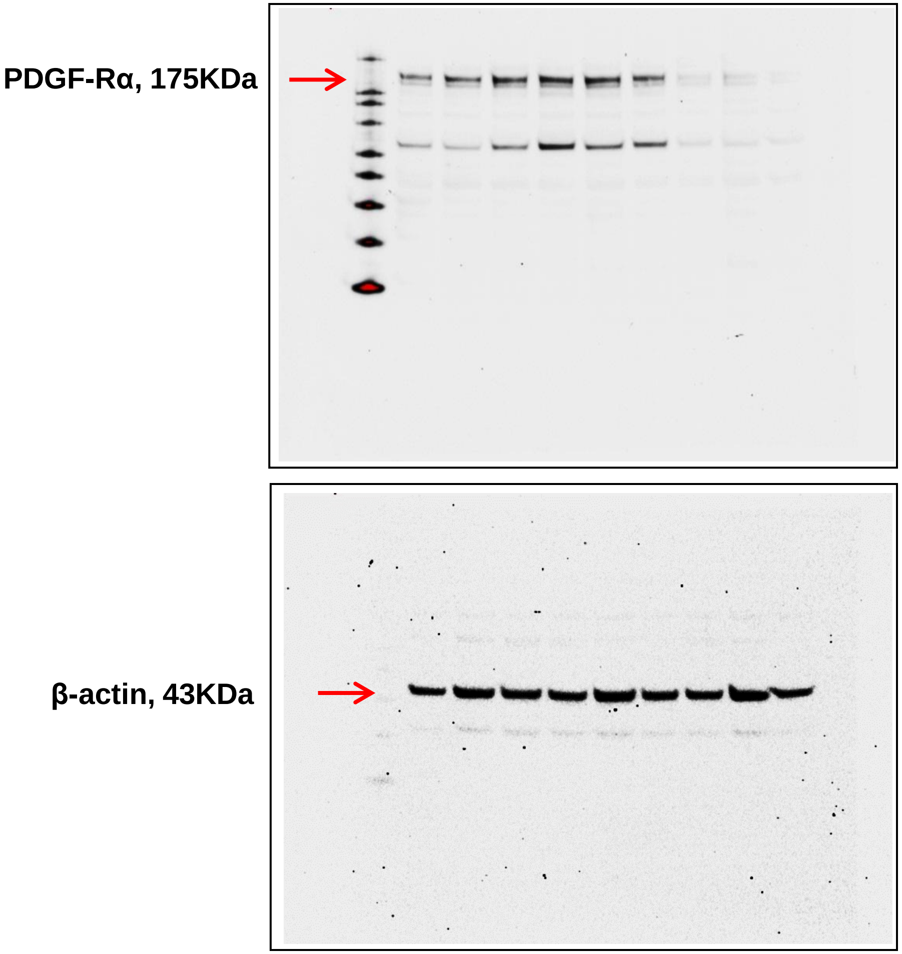

Figure 6H

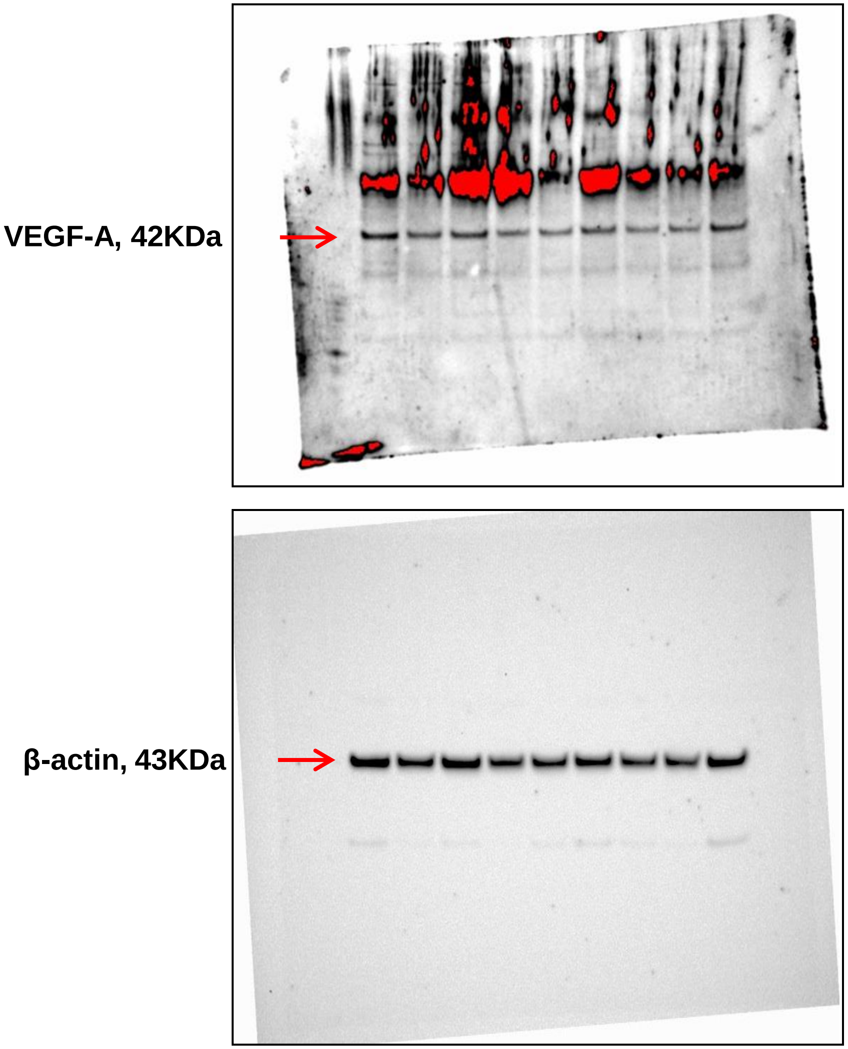

Figure 6I

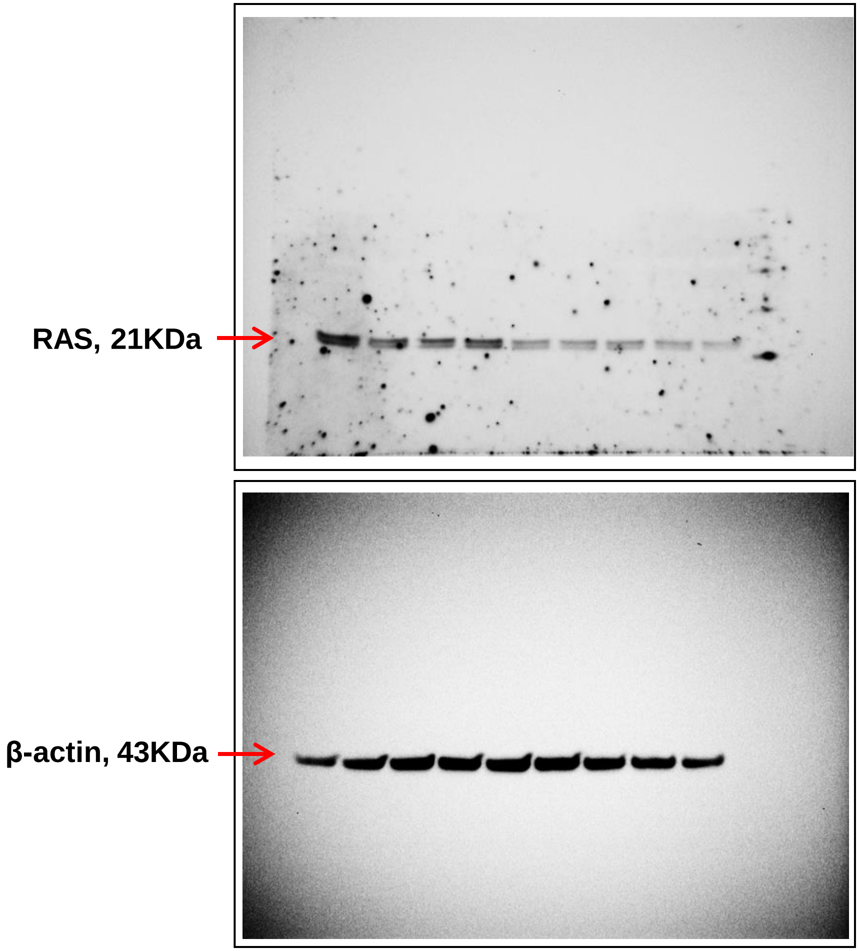

Figure 6J

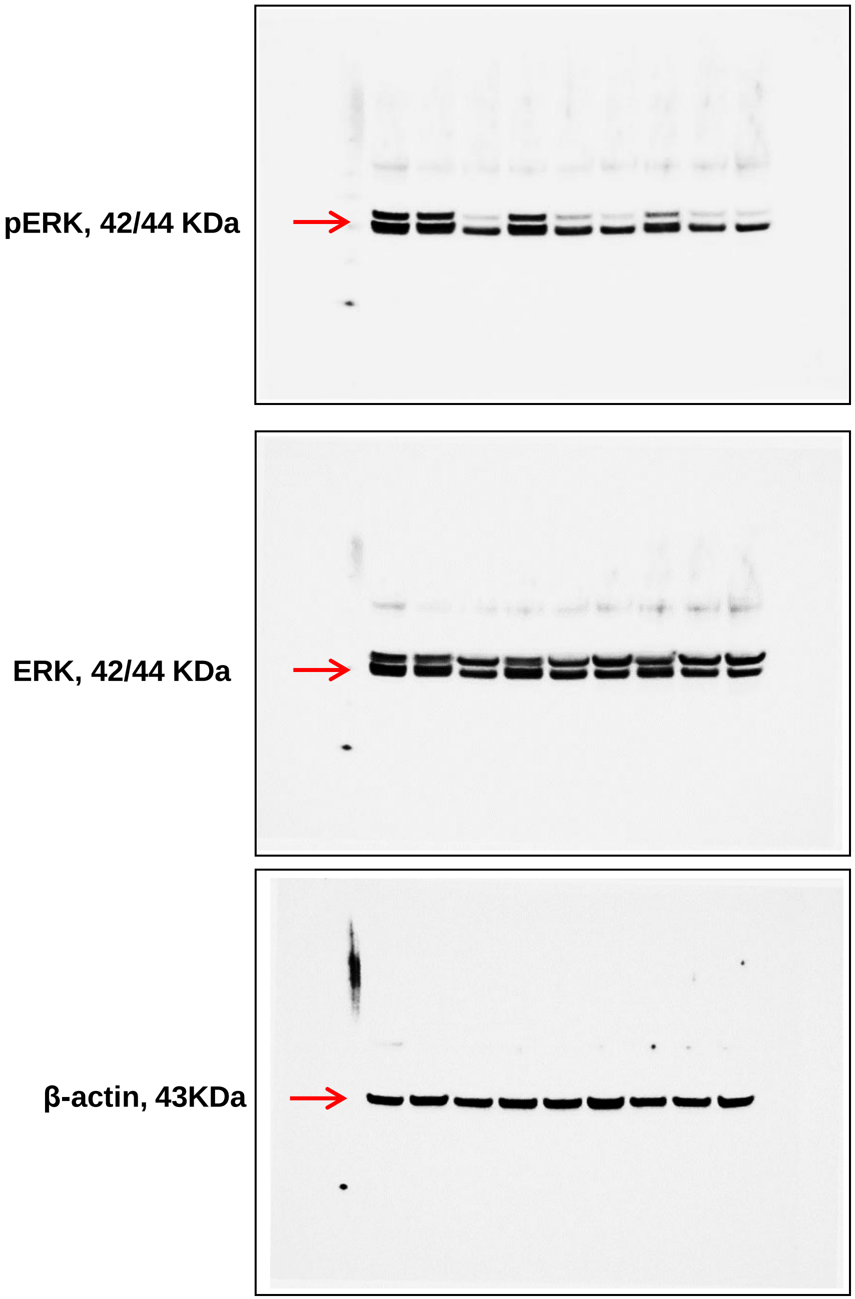

Figure 6L

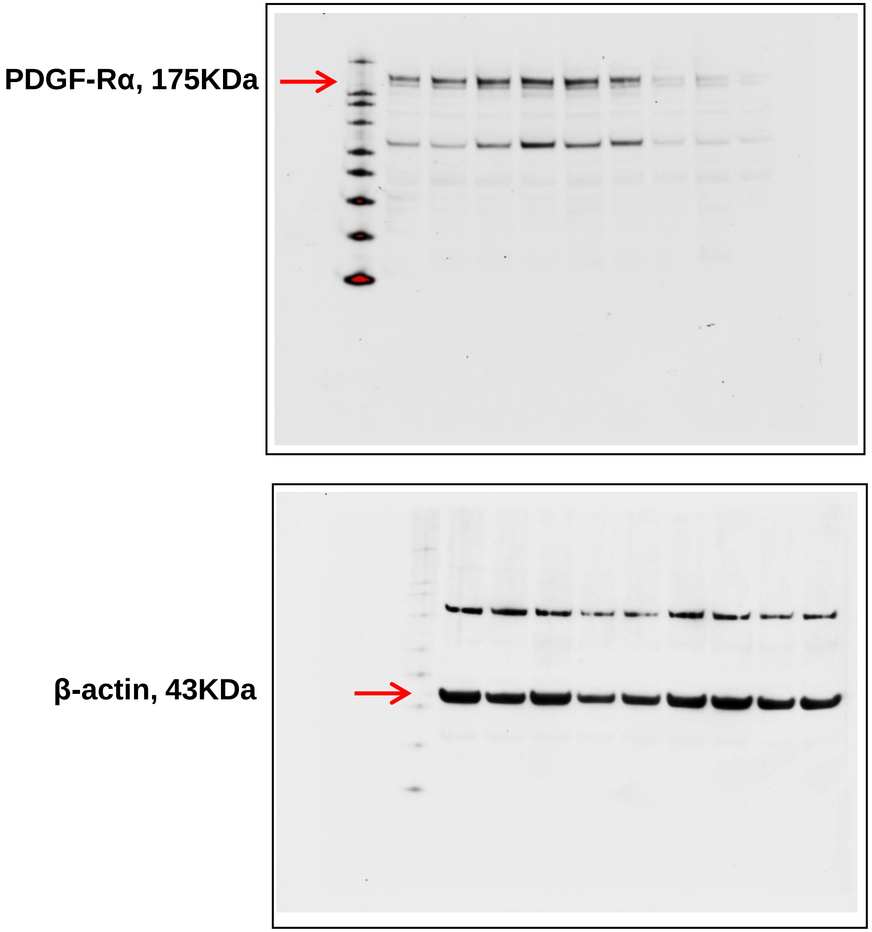

Supplement: Supplementary file 10 — Source Data for Figure 6 [file EMMM-9-1504-s008.pdf]
